# Hierarchical Bi_2_WO_6_/TiO_2_-nanotube composites derived from natural cellulose for visible-light photocatalytic treatment of pollutants

**DOI:** 10.3762/bjnano.13.66

**Published:** 2022-08-04

**Authors:** Zehao Lin, Zhan Yang, Jianguo Huang

**Affiliations:** 1 Department of Chemistry, Zhejiang University, Hangzhou, Zhejiang 310027, P. R. China; 2 Shenzhen Middle School, Shenzhen, Guangdong 518001, P. R. China

**Keywords:** biomimetic synthesis, cellulose, nanoarchitectonics, nanocomposite, nanotubes, photocatalysis, pollutants

## Abstract

A series of Bi_2_WO_6_/TiO_2_-nanotube (Bi_2_WO_6_/TiO_2_-NT) heterostructured composites were prepared by utilizing natural cellulose (e.g., laboratory filter paper) as the structural template. The obtained nanoarchitectonics, namely Bi_2_WO_6_/TiO_2_-NT nanocomposites, displayed three-dimensionally interwoven structures which replicated the initial cellulose template. The composite Bi_2_WO_6_/TiO_2_-NT nanotubes were formed by TiO_2_ nanotubes that uniformly anchored with Bi_2_WO_6_ nanoparticles of various densities on the surface. The composites exhibited improved photocatalytic activities toward the reduction of Cr(VI) and degradation of rhodamine B under visible light (λ > 420 nm), which were attributed to the uniform anchoring of Bi_2_WO_6_ nanoparticles on TiO_2_ nanotubes, as well as strong mutual effects and well-proportioned formation of heterostructures in between the Bi_2_WO_6_ and TiO_2_ phases. These improvements arose from the cellulose-derived unique structures, leading to an enhanced absorption of visible light together with an accelerated separation and transfer of the photogenerated electron–hole pairs of the nanocomposites, which resulted in increased effective amounts of photogenerated carriers for the photocatalytic reactions. It was demonstrated that the photoinduced electrons dominated the photocatalytic reduction of Cr(VI), while hydroxyl radicals and reactive holes contributed to the photocatalytic degradation of rhodamine B.

## Introduction

The direct emission of untreated pollutants including dyes, organic matter, and heavy metals has caused disastrous consequences to the ecosystem [1]. Cr(VI) is one of the unmanageable pollutants in industrial effluents. It is highly toxic, carcinogenic, and harmful to the lungs, liver, and other organs [2–3]. Photocatalysis is a promising method for the reduction of Cr(VI) into Cr(III) due to its high efficiency, energy-saving, and nonpolluting advantages [4]. Among the various photocatalysts, traditional titania (TiO_2_) photocatalysts have received great attention due to their high reactivity, excellent stability, and nontoxicity [5–8]. However, the wide bandgap of TiO_2_ inhibits the absorption of light in the visible region and the rapid recombination of photogenerated electron−hole pairs restrains its photocatalytic activity. It has been verified that constructing TiO_2_-based heterostructured composites by using visible-light-responsive semiconductors with suitable band structures provides a pathway for the advancement of highly efficient photocatalysts [9]. It has been demonstrated that the assembly of various nanoscale building blocks to form the corresponding nanoarchitectonics provides an ideal pathway for the syntheses of a large variety of functional materials [10–15]; in particular, for the fabrication of specific catalytic materials [16–19].

Recently, several bismuth-based photocatalysts have drawn extensive attention owing to their unique band structures and excellent stability against photocorrosion [20]. Among them, the Aurivillius phase bismuth tungstate (Bi_2_WO_6_) material is applied in visible-light photocatalysis due to its effective response to visible light and stable physicochemical properties [21]. Bi_2_WO_6_ is formed by the alternating growth of (Bi_2_O_2_)^2+^ and perovskite-structured (WO_4_)^2−^ units [22]. The hybridized O 2p and Bi 6s orbitals in the conduction band of the material contribute to the effective transfer of photoinduced electron−hole pairs, resulting in good photocatalytic properties under visible-light irradiation [23–24]. However, there is still room for improvement of the photocatalytic performance of the Bi_2_WO_6_ material since the recombination of photogenerated charges during photocatalysis is still too fast to produce a large amount of effective photoinduced carriers [25].

In order to solve the aforementioned problems of TiO_2_ and Bi_2_WO_6_ materials, researchers have fabricated some Bi_2_WO_6_/TiO_2_ composites, which were employed in various photocatalytic applications, such as degradation of organic pollutants [25], oxidation of methane [24], and production of hydrogen by water splitting [26]. According to these reports, Bi_2_WO_6_/TiO_2_ composites have better photocatalytic activities than those of pure TiO_2_ and Bi_2_WO_6_. This is ascribed to the heterostructures built in between the two phases, leading to accelerated separation and transfer of photogenerated electrons and holes. Nevertheless, the effective formation of heterostructures in between the TiO_2_ and Bi_2_WO_6_ phases are limited owing to the aggregation of these phases, resulting in the decrement of active sites during photocatalysis [27]. In order to further increase the effectiveness of these heterostructures, the fabrication of efficient TiO_2_ materials for a homogeneous dispersion of Bi_2_WO_6_ through its morphological control is expected to offer a solution [28].

Based on biomimetic synthesis, the morphological control of functional materials is accomplished by combining chemical building blocks and natural substances with unique structures. Natural cellulose products (e.g., filter paper for quantitative analysis) possess the advantages of being abundant, environmentally friendly and biocompatible, and the functional hydroxyl groups on the surface provide a chemical environment for the deposition of the guest components [29–30]. According to our results, the composite photocatalysts synthesized by employing natural cellulose as the template had enhanced the photocatalytic activities. This was due to the uniform and stable integration among their components, which is mainly attributed to the hierarchical micro-to-nanoscale structure replicated from the original cellulose template [31–33]. This structural bionics procedure using natural cellulose can be used in the fabrication of Bi_2_WO_6_/TiO_2_ composites. However, the relationship between the structure and activity of these compounds still remain to be deeply investigated.

In this work, cellulose-derived Bi_2_WO_6_/TiO_2_-NT heterostructured composites were fabricated by depositing Bi_2_WO_6_ nanoparticles on hierarchically interwoven TiO_2_ nanotubes via the solvothermal method, and the densities of Bi_2_WO_6_ nanoparticles in the composites were regulated by the concentrations of the precursors. When the Bi_2_WO_6_/TiO_2_-NT nanocomposites were used for the photocatalytic reduction of Cr(VI) or degradation of rhodamine B (RhB) under visible-light irradiation (λ > 420 nm), they exhibited excellent photocatalytic activities. This was due to the unique hierarchical network structures of the composite as well as the uniformly and compactly built heterostructures in between TiO_2_ and Bi_2_WO_6_ phases. Meanwhile, the relationships between structure and activity of the nanocomposite, reasons for the enhancement of the photocatalytic performances, and possible photocatalytic mechanism of Bi_2_WO_6_/TiO_2_-NT nanocomposites were explored and discussed.

## Experimental

### Chemicals

Toluene, anhydrous ethanol, acetone, barium sulfate (BaSO_4_), rhodamine B, isopropyl alcohol (IPA), *N*-methylpyrrolidone, ethylenediaminetetraacetic acid disodium salt (EDTA-2Na), silver nitrate (AgNO_3_), sodium sulfate (Na_2_SO_4_), ethylene glycol (EG), potassium dichromate (K_2_Cr_2_O_7_), phosphoric acid (H_3_PO_4_), and sulfuric acid (H_2_SO_4_) were purchased from Sinopharm Chemical Reagent Co., Ltd. (China). Titanium *n*-butoxide [Ti(O*^n^*Bu)_4_], *p*-benzoquinone (*p*-BQ), bismuth nitrate pentahydrate [Bi(NO_3_)_3_·5H_2_O], sodium tungstate dihydrate (Na_2_WO_4_·2H_2_O), 1,5-diphenylcarbazide, and potassium iodate (KIO_3_) were bought from J&K Scientific Ltd. Polyvinylidene difluoride (PVDF) was obtained from Fluorochem Co., Ltd. All chemicals were directly applied without further treatment. Filter paper for quantitative laboratory analysis (made from cotton) was purchased from Hangzhou Xinhua Paper Industry Co., Ltd. (China). Ultrapure water was obtained from the Milli-Q Advantage A 10 system (Millipore, Bedford, MA, USA), displaying a resistivity of 18.2 MΩ·cm.

### Preparation of Bi_2_WO_6_/TiO_2_-NT nanocomposites

**Figure 1:**
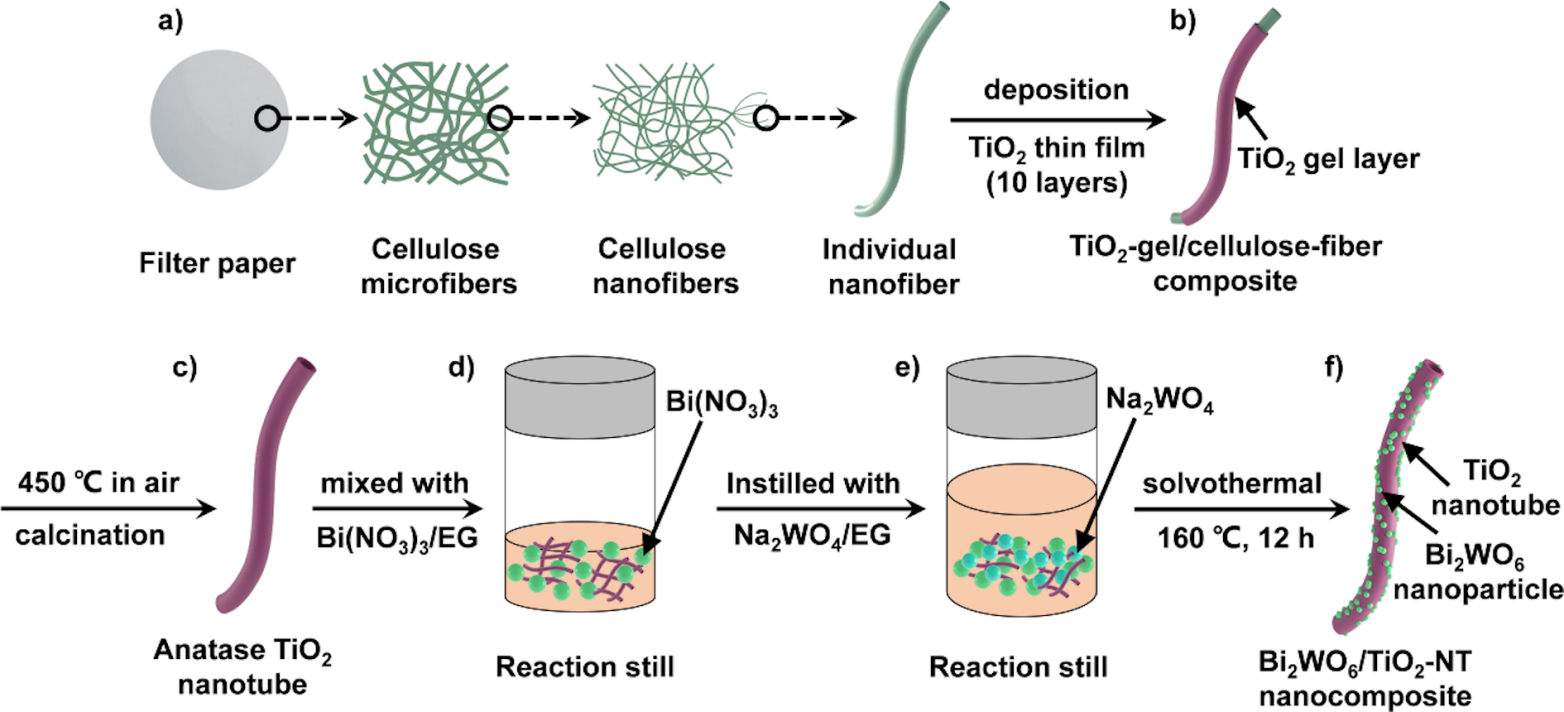
Schematic illustration of the fabrication process of cellulose-derived Bi_2_WO_6_/TiO_2_-NT nanocomposites.

The fabrication process of cellulose-derived Bi_2_WO_6_/TiO_2_-NT nanocomposites is exhibited in Figure 1. Ten layers of ultrathin titania film were deposited onto cellulose nanofiber templates by the surface sol−gel method according to a previous report (Figure 1a, Figure 1b) [31]. The as-prepared TiO_2_-gel/cellulose-fiber composite was calcined in air at 450 °C (heating rate: 1 °C/min) for 6 h to give the hierarchical structure of TiO_2_ nanotubes (Figure 1c).

Subsequently, Bi_2_WO_6_/TiO_2_-NT nanocomposites were prepared by the solvothermal method with hierarchical TiO_2_ nanotubes (40.0 mg), Bi(NO_3_)_3_·5H_2_O, and Na_2_WO_4_·2H_2_O as raw materials. According to the theoretical contents of Bi_2_WO_6_ components in the Bi_2_WO_6_/TiO_2_-NT nanocomposites (30, 50, 70, and 90 wt %), the dosages of the Bi(NO_3_)_3_·5H_2_O and Na_2_WO_4_·2H_2_O reagents were calculated and presented in Supporting Information File 1, Table S1. The corresponding nanocomposites were labelled as 30%−Bi_2_WO_6_/TiO_2_-NT, 50%−Bi_2_WO_6_/TiO_2_-NT, 70%−Bi_2_WO_6_/TiO_2_-NT, and 90%−Bi_2_WO_6_/TiO_2_-NT, respectively. For instance, Bi(NO_3_)_3_·5H_2_O (129.7 mg) was dissolved in EG (20.0 mL) and mixed with the hierarchical TiO_2_ nanotubes (40.0 mg), which were stirred for 2 h (Figure 1d). Then, Na_2_WO_4_·2H_2_O (44.1 mg) was dissolved in EG (20.0 mL), instilled into the aforementioned mixture, and stirred for 3 h (Figure 1e). The solvothermal reaction was conducted at 160 °C (heating rate: 5 °C/min) for 12 h, and the obtained powder sample was washed several times with ethanol and then dried at 37 °C in vacuum, yielding the 70%−Bi_2_WO_6_/TiO_2_-NT nanocomposite (Figure 1f).

### Preparation of control materials

In order to further investigate the photocatalytic mechanism and performances of cellulose-derived Bi_2_WO_6_/TiO_2_-NT nanocomposites, pure TiO_2_ nanotubes (TiO_2_-NT), pure Bi_2_WO_6_ powder, Bi_2_WO_6_/TiO_2_, and Bi_2_WO_6_-TiO_2_ were prepared as contrast materials. The hierarchical TiO_2_ nanotubes derived from natural cellulose were regarded as the pure TiO_2_-NT sample without any of the subsequent treatments. The Bi_2_WO_6_ powder sample was fabricated by the same solvothermal reaction without the addition of the powder containing hierarchical TiO_2_ nanotubes. The TiO_2_ sample without the cellulose template was prepared by the simple sol−gel method with the Ti(O*^n^*Bu)_4_ solution (100.0 mM in ethanol/toluene, v/v = 1:1) as the precursor, followed by the calcination process at 450 °C for 6 h. Then, the Bi_2_WO_6_/TiO_2_ sample with a theoretical Bi_2_WO_6_ content of 70 wt % (without the cellulose template) was fabricated by the same solvothermal reaction method and conditions with the 70%−Bi_2_WO_6_/TiO_2_-NT nanocomposite, and the hierarchical TiO_2_ nanotubes were replaced by the TiO_2_ sample without the cellulose template. The Bi_2_WO_6_-TiO_2_ sample was prepared by the physical mixing of pure TiO_2_ nanotubes (27.1 mg) and pure Bi_2_WO_6_ powder (72.9 mg) according to the practical content (72.9 wt %) of the Bi_2_WO_6_ component in the 70%−Bi_2_WO_6_/TiO_2_-NT nanocomposite.

### Characterization

Powder X-ray diffraction (XRD) patterns of the samples were obtained from the Rigaku Ultima IV diffractometer with a Cu Kα (λ = 0.15405 nm) radiation source. Fourier transform infrared (FTIR) spectra of the samples were recorded on the Nicolet iS10 spectrometer. The X-ray photoelectron spectroscopy (XPS) experiment was performed on the Thermo Scientific ESCALAB 250Xi spectrometer equipped with an Al Kα X-ray source at an energy value of 1486.6 eV. The XPS spectra were calibrated by using a reference to the peak of the surface adventitious carbon (284.8 eV) in the high-resolution spectrum of the C 1s region. The N_2_ adsorption−desorption isotherms were recorded at −196 °C on a Micromeritics ASAP 2020 analyzer, while the specific surface area and pore distribution curve were determined according to the Brunauer−Emmett−Teller (BET) model and the Barrett−Joyner−Halenda (BJH) method. The UV–visible diffuse reflectance spectra (UV–vis DRS) were acquired on a Shimadzu UV-2450 spectrophotometer in the diffuse reflectance mode, equipped with an integrating sphere and using BaSO_4_ as the reference standard for solid samples. The photoluminescence (PL) spectra were obtained on a Shimadzu RF-5301PC fluorescence spectrometer under a laser excitation of 360 nm.

In order to observe the microstructures of the samples, a small amount of a given sample was dispersed in ethanol to generate a homogeneous suspension. The suspension was then dropped onto an Al foil to be observed via field-emission scanning electron microscopy (FE-SEM), and onto a carbon-supported copper grid for examination via transmission electron microscopy (TEM) and high resolution transmission electron microscopy (HR-TEM). Meanwhile, selected area electron diffraction (SAED) patterns and energy dispersive X-ray spectrometry (EDX) mapping images were obtained via HR-TEM. The instrument models used in FE-SEM, TEM, and HR-TEM were Hitachi SU-8010, Hitachi HT-7700, and JEM-2100F, and the working voltages were 5.0, 100, and 200 kV, respectively.

The transient photocurrent responses and electrochemical impedance spectroscopy (EIS) Nyquist plots (frequency: 0.01 Hz−100 kHz, alternate current: 5 mV) of a given sample were obtained on a CHI 760D (Shanghai, China) electrochemical workstation using a three-electrode system. The Pt plate (1.0 × 1.0 cm^2^) and saturated calomel electrode (SCE) were used as the reference and counter electrodes, respectively. The Na_2_SO_4_ aqueous solution (0.5 M) was employed as the electrolyte and a xenon lamp with a 420 nm cutoff filter was used as the light source. The corresponding sample (5.0 mg) was ultrasonically dispersed in ethanol (1.0 mL) with the addition of a PVDF solution (100.0 μL, 10.0 mg·mL^−1^, using *N*-methylpyrrolidone as the solvent), which was then spin-coated on a conducting indium tin oxide (ITO) glass, followed by the calcination process at 100 °C for 24 h, giving the relevant working electrode.

### Photocatalytic experiments

The photocatalytic experiments were conducted on the XPA-1 photoreactor (Nanjing Xujiang, China) under a constant temperature (20 °C), and a 350 W xenon lamp with a 420 nm cutoff filter was employed as the visible-light source, which was kept in a horizontal distance of 5.0 cm from the quartz tube reactor. The photocatalyst (10.0 mg) was dispersed in the pollutant solution (20.0 mL, 10.0 mg·L^−1^) and stirred in the dark for 1 h to guarantee the adsorption−desorption equilibrium. Then, the visible light was switched on, and the suspension containing photocatalyst and pollutant solution was taken out at set intervals to determine the concentration variation of the pollutant solution.

Cr(VI) and RhB were chosen as pollutant representatives, and the concentration of the RhB aqueous solution was measured by the absorbance at λ = 553 nm, analyzed through a Shimadzu UV-2450 spectrophotometer. The concentration of the Cr(VI) aqueous solution was determined by the diphenylcarbazide colorimetric method as follows. 1,5-diphenylcarbazide (0.2 g) was dissolved in acetone/water (100.0 mL, v/v = 1:1) to form solution A, while H_3_PO_4_ (20.0 mL) and H_2_SO_4_ (20.0 mL) were homogeneously mixed in water (40.0 mL) to form solution B. The Cr(VI) pollutant solution (1.0 mL), solution A (40.0 μL), and solution B (20.0 μL) were uniformly blended for 6 min, and the absorbance at λ = 540 nm of the mixed solution was recorded by a Shimadzu UV-2450 spectrophotometer to give the concentration of the corresponding Cr(VI) pollutant solution. In addition, the pH values (2, 3, 4, and 5) of the initial Cr(VI) pollutant solution were adjusted using the H_2_SO_4_ (0.1 M) aqueous solution. In order to obtain the photocatalytic performances of the samples, the removal percentage of Cr(VI) and degradation percentage of RhB were determined by Equation 1, while the apparent reaction rates (*K*_app_) of the corresponding photocatalytic reactions were determined by Equation 2, according to the pseudo-first-order kinetic model:


[1] $$\text{Removal}\left(\text{degradation}\right)\text{percentage}=\left[\left(C-C_{i}\right)/C_{i}\right]\times 100\%,$$

[2] $$\ln\left(C_{0}/C\right)=K_{\text{app}}\times t,$$

where *C*, *C*_0_ and *C_i_* represent the concentrations of the Cr(VI) or RhB pollutant solutions after irradiation for *t* hours, after the achievement of the adsorption−desorption equilibrium, and at the initial moment (10.0 mg·L^−1^), respectively.

The photocatalytic stabilities of the cellulose-derived Bi_2_WO_6_/TiO_2_-NT nanocomposites on the photocatalytic reduction of Cr(VI) or degradation of RhB were confirmed by using the 70%−Bi_2_WO_6_/TiO_2_-NT nanocomposite. After the photocatalytic reaction of the first cycle, the photocatalyst was separated from the pollutant solution, washed with ethanol, and dried at 37 °C under vacuum for 12 h. After that, the photocatalyst was applied to the second cycle of the photocatalytic reaction under the same conditions, and the cyclic reactions were carried out for five times in total.

The 70%−Bi_2_WO_6_/TiO_2_-NT nanocomposite was also employed to explore the photocatalytic mechanism as the representative of the Bi_2_WO_6_/TiO_2_-NT nanocomposites. Before the photocatalytic reduction of Cr(VI), IPA (0.1 M), EDTA-2Na (10.0 mM), KIO_3_ (0.1 M), and AgNO_3_ (0.1 M) were added into the reaction solution to shield the hydroxyl radicals (^•^OH), reactive holes (h^+^), superoxide radicals (^•^O_2_^−^) and electrons (e^−^) species, respectively, generated during the photocatalysis. Similarly, IPA (0.1 M), EDTA-2Na (0.1 M), and p-BQ (5.0 mM) were also put into the initial RhB pollutant solution to capture ^•^OH, h^+^, and ^•^O_2_^−^ species, respectively, generated during the photocatalytic degradation of RhB.

## Results and Discussion

### Structural characterization

As shown in Figure 1, the Bi_2_WO_6_/TiO_2_-NT nanocomposites were fabricated by depositing Bi_2_WO_6_ nanoparticles onto the hierarchical TiO_2_ tubes via the solvothermal method, where the TiO_2_ tubes were obtained by the calcination of the TiO_2_-gel/cellulose composite, which in turn was prepared through the surface sol−gel method. According to the theoretical contents (30, 50, 70, and 90 wt %) of the Bi_2_WO_6_ component in the Bi_2_WO_6_/TiO_2_-NT nanocomposites, they were denoted as 30%−Bi_2_WO_6_/TiO_2_-NT, 50%−Bi_2_WO_6_/TiO_2_-NT, 70%−Bi_2_WO_6_/TiO_2_-NT, and 90%−Bi_2_WO_6_/TiO_2_-NT nanocomposites, respectively. The practical contents of the Bi_2_WO_6_ component in these Bi_2_WO_6_/TiO_2_-NT nanocomposites were measured by EDX to be 38.4, 54.3, 72.9, and 95.2 wt %, respectively, as displayed in Supporting Information File 1, Figure S1 and Table S2.

**Figure 2:**
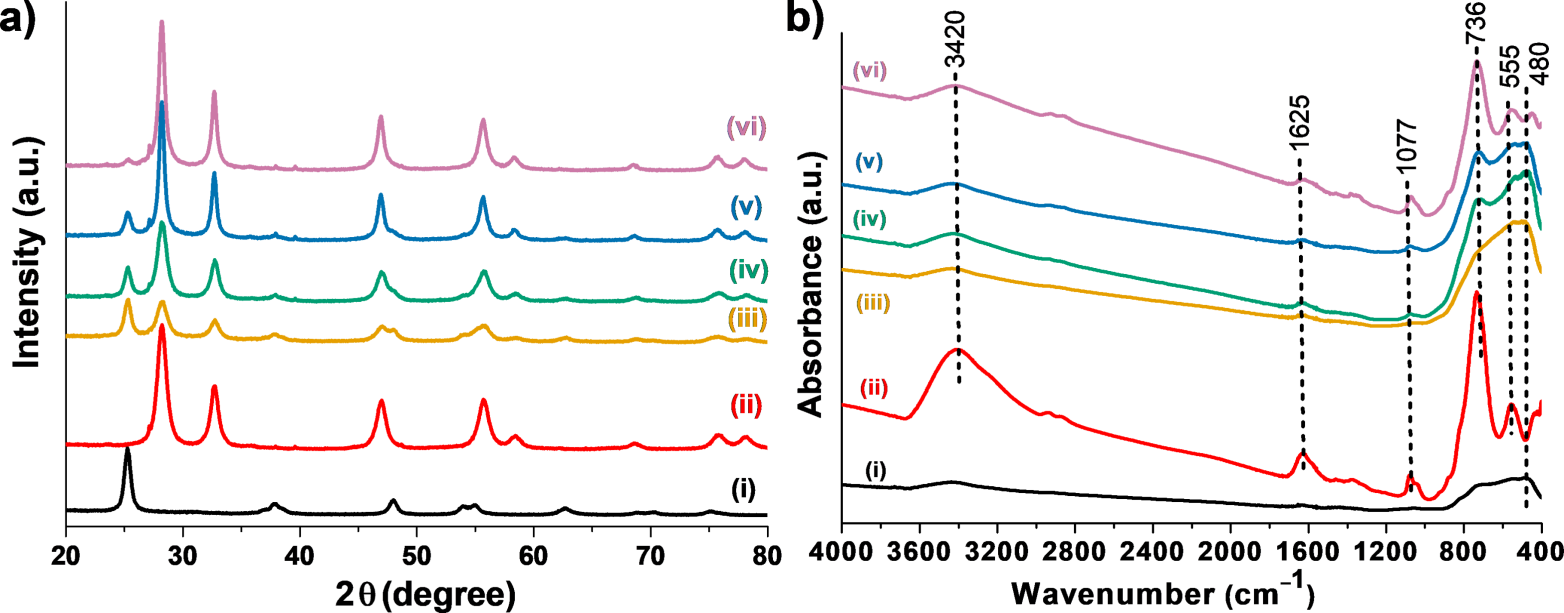
(a) XRD patterns and (b) FTIR spectra of (i) pure TiO_2_-NT and (ii) pure Bi_2_WO_6_ samples, as well as of hierarchical (iii) 30%−Bi_2_WO_6_/TiO_2_-NT, (iv) 50%−Bi_2_WO_6_/TiO_2_-NT, (v) 70%−Bi_2_WO_6_/TiO_2_-NT, and (vi) 90%−Bi_2_WO_6_/TiO_2_-NT nanocomposites.

As shown in Figure 2a, the XRD patterns of the Bi_2_WO_6_/TiO_2_-NT nanocomposites all exhibit characteristic diffraction peaks at 2θ = 28.3, 32.7, 47.1, 55.9, 58.5, 68.6, 75.7, and 78.2°, which are attributed to the (131), (200), (202), (133), (262), (400), (391), and (460) planes of the russellite phase Bi_2_WO_6_ (JCPDS No. 39-0256), respectively [34]. Besides, the peak located at 2θ = 25.3° is also observed in these XRD patterns, which is assigned to the (101) plane of the anatase phase titania (JCPDS No. 21-1272) [34]. The diffraction peaks in the XRD pattern of pure Bi_2_WO_6_ powder are all consistent with those of the Bi_2_WO_6_/TiO_2_-NT nanocomposites and assigned to the russellite phase Bi_2_WO_6_. The XRD pattern of pure TiO_2_-NT shows other weak peaks at 2θ = 37.8, 48.0, 53.9, 55.1, 65.7, and 75.0°, which belong to the (004), (200), (105), (211), (204), and (215) planes of the anatase phase titania (JCPDS No. 21-1272), respectively [34]. These weak peaks are only presented in the XRD pattern of the 30%−Bi_2_WO_6_/TiO_2_-NT nanocomposite due to the rather low contents of the TiO_2_ component in other Bi_2_WO_6_/TiO_2_-NT nanocomposites. Besides, the intensities of the characteristic diffraction peaks in the XRD patterns of the Bi_2_WO_6_/TiO_2_-NT nanocomposites weaken with the increased content of the TiO_2_ component in the corresponding Bi_2_WO_6_/TiO_2_-NT nanocomposite. The presence of the hierarchical TiO_2_ nanotubes influences and inhibits the crystallinity of Bi_2_WO_6_ in between the interface of the two phases, revealing the strong interaction between TiO_2_ and Bi_2_WO_6_ phases [35–36].

Figure 2b displays the FTIR spectra of the hierarchical Bi_2_WO_6_/TiO_2_-NT nanocomposites, pure TiO_2_-NT, and pure Bi_2_WO_6_ powder samples, where all present two similar absorption bands at 1625 and 3420 cm^−1^ which can be indexed to the stretching vibration of adsorbed H_2_O and –OH group on the sample surface [37]. Apart from the 30%−Bi_2_WO_6_/TiO_2_-NT nanocomposite, the FTIR spectra of the other Bi_2_WO_6_/TiO_2_-NT nanocomposites all exhibit three apparent absorption bands at approx. 555, 736, and 1077 cm^−1^, which are indexed to the stretching vibrations of Bi−O and W−O covalent bonds and to the bridge stretching vibration of the W−O−W bond in the Bi_2_WO_6_ phase, respectively [38]. All the FTIR spectra of the Bi_2_WO_6_/TiO_2_-NT nanocomposites display bands centered at approx. 480 cm^−1^ which are assigned to the Ti−O stretching vibration in the TiO_2_ phase except for the 90%−Bi_2_WO_6_/TiO_2_-NT nanocomposite [39]. The FTIR spectrum of the pure Bi_2_WO_6_ powder exhibits similar absorption bands to those of the 90%−Bi_2_WO_6_/TiO_2_-NT nanocomposite, which are all attributed to the Bi_2_WO_6_ phase. In the spectrum of pure TiO_2_-NT, apart from the band indexed to the –OH group (3420 cm^−1^), another wide band centered at 600 cm^−1^ is detected which is assigned to the Ti−O stretching vibration in the TiO_2_ phase. As compared to the spectra of pure TiO_2_-NT and pure Bi_2_WO_6_ powder samples, the absorption bands in the Bi_2_WO_6_/TiO_2_-NT nanocomposites all exhibit slight red shifts, demonstrating the close connection, strong interaction, and formation of heterostructures in between Bi_2_WO_6_ and TiO_2_ phases. It is deduced from the XRD and FTIR characterizations that the Bi_2_WO_6_/TiO_2_-NT nanocomposites are only composed of the anatase phase TiO_2_ and the russellite phase Bi_2_WO_6_, while strong mutual effects and well-proportioned heterostructures are organized in between the two phases.

**Figure 3:**
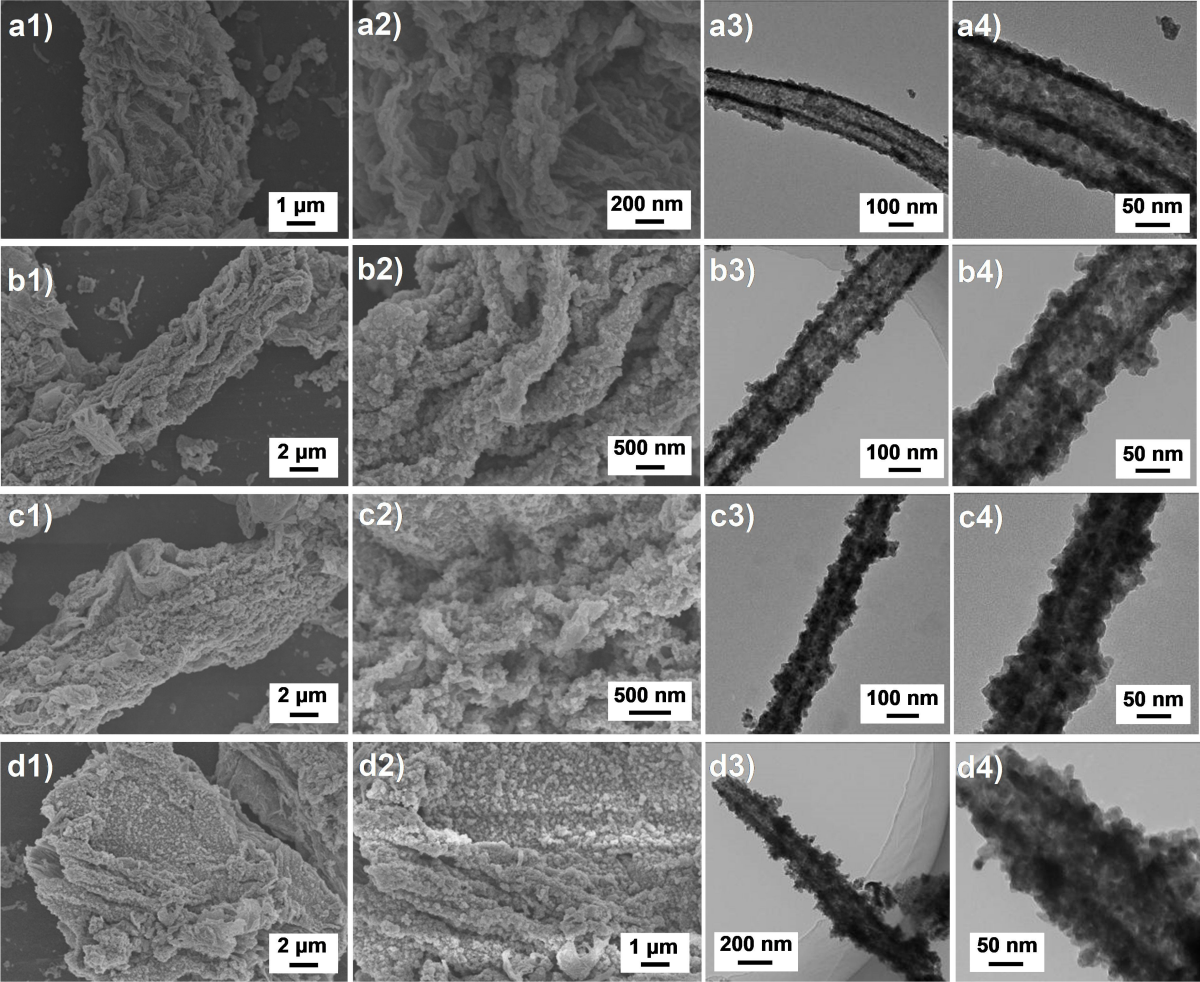
Electron micrographs of the hierarchical (a1−a4) 30%−Bi_2_WO_6_/TiO_2_-NT, (b1−b4) 50%−Bi_2_WO_6_/TiO_2_-NT, (c1−c4) 70%−Bi_2_WO_6_/TiO_2_-NT, and (d1−d4) 90%−Bi_2_WO_6_/TiO_2_-NT nanocomposites. The first two columns represent FE-SEM images of the nanocomposites showing the three-dimensional network structures. The last two columns exhibit TEM images of an individual composite nanotube which was separated from the corresponding nanocomposite.

Figure 3 presents the morphologies and microstructures of cellulose-derived Bi_2_WO_6_/TiO_2_-NT nanocomposites. As exhibited in the FE-SEM images (the first two columns in Figure 3), all Bi_2_WO_6_/TiO_2_-NT nanocomposites are assembled by composite microtubes, which are composed of cross-linked nanotubes, revealing the hierarchical network structures replicated from the initial cellulose template. Besides, it is apparent that the uniform Bi_2_WO_6_ nanoparticles are compactly coated on TiO_2_ nanotubes. The TEM images (the last two columns in Figure 3) of individual composite nanotubes isolated from the Bi_2_WO_6_/TiO_2_-NT nanocomposites show similar nanotube diameters of approx. 100 nm, and the uniform Bi_2_WO_6_ nanoparticles (sizes: 10−20 nm) are compactly coating the TiO_2_ nanotubes which are composed of tiny TiO_2_ nanocrystallites. With an increased Bi_2_WO_6_ content in the Bi_2_WO_6_/TiO_2_-NT nanocomposites, the thicknesses of the isolated nanotubes increase in the order of 15, 25, 40 and 60 nm, and the densities of uniform Bi_2_WO_6_ nanoparticles on the TiO_2_ nanotubes also increase.

As a comparison, the pure TiO_2_-NT sample retains the three-dimensional network structure of natural cellulose, and the TiO_2_ nanotube has a tube diameter of 100 nm and a thickness of 5 nm (Supporting Information File 1, Figure S2a), proving that the deposition of Bi_2_WO_6_ nanoparticles does not affect the hierarchically cross-linked structures but thickens the composite nanotubes of the Bi_2_WO_6_/TiO_2_-NT nanocomposites. The pure Bi_2_WO_6_ powder sample is formed by aggregated Bi_2_WO_6_ particles with sizes of 10−20 nm (Figure S2b). The Bi_2_WO_6_/TiO_2_ sample that was prepared without the cellulose template is composed of several aggregated Bi_2_WO_6_ nanoparticles on the bulk TiO_2_, while the distribution of Bi_2_WO_6_ nanoparticles is rather uneven with very different sizes (Figure S2c). It is hence concluded that the cellulose-derived three-dimensional network structures of the Bi_2_WO_6_/TiO_2_-NT nanocomposites promote the uniform dispersion of the Bi_2_WO_6_ nanoparticles on the TiO_2_ nanotubes, which is beneficial to the formation of active sites and well-proportioned heterostructures for the photocatalytic reactions. As illustrated in Figure 1, when the hierarchical TiO_2_ nanotubes were mixed with the precursor solutions of Bi_2_WO_6_, the Bi^3+^ ions were uniformly dispersed on the negatively charged tube surfaces due to its unique morphology, which resulted in more uniform formation of the Bi_2_WO_6_ nanoparticles in the Bi_2_WO_6_/TiO_2_-NT nanocomposites. The detailed mechanism was revealed in the hierarchical Ag_2_O-nanoparticle/TiO_2_-nanotube composite reported by our group [31].

**Figure 4:**
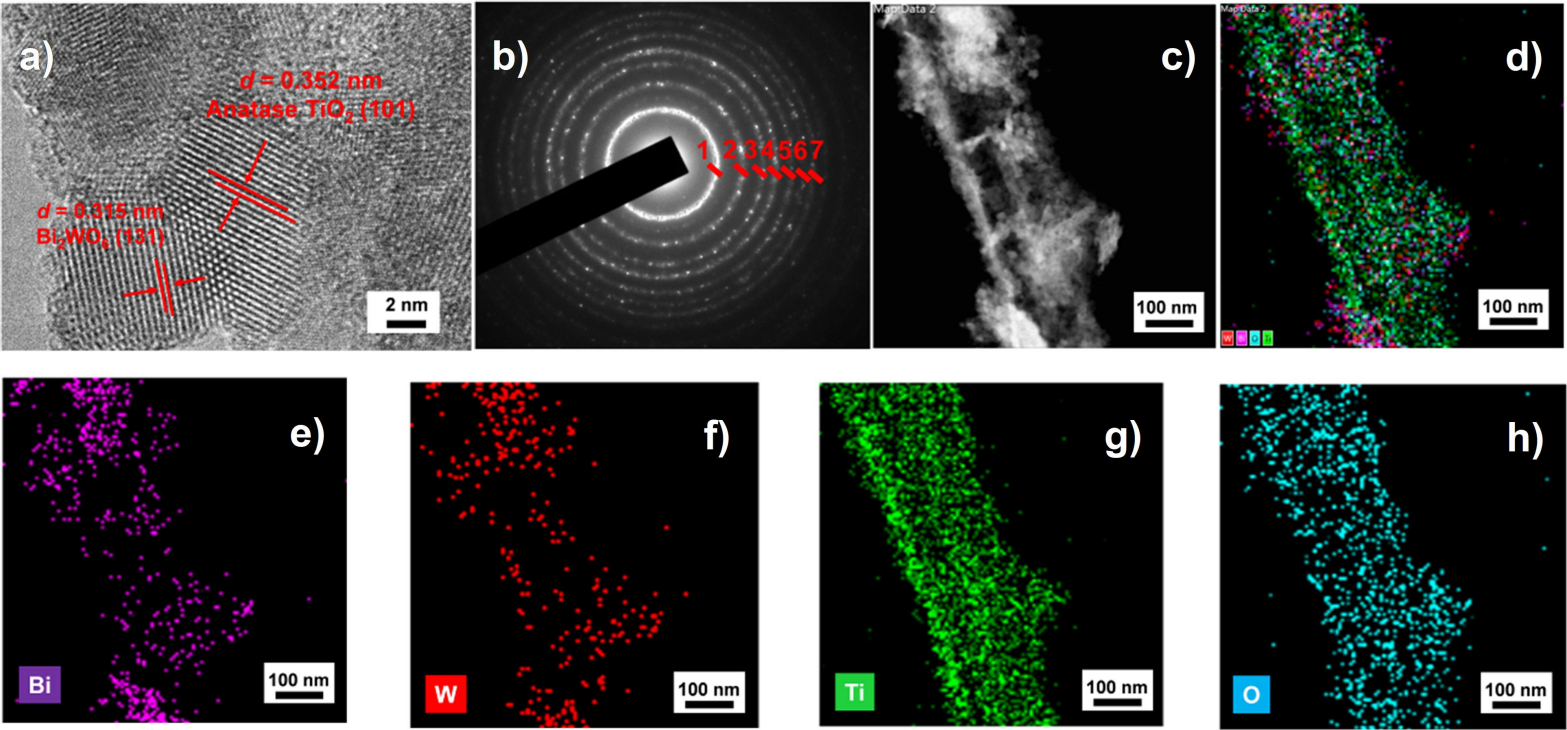
(a) HR-TEM image, (b) SAED pattern, and (c−h) EDX element mapping images of Bi, W, Ti, and O elements of the composite nanotube surface in the hierarchical 70%−Bi_2_WO_6_/TiO_2_-NT nanocomposite.

As shown in Figure 4a, the HR-TEM image of the 70%−Bi_2_WO_6_/TiO_2_-NT nanocomposite shows two kinds of lattice fringes with interplanar spacings of 0.315 and 0.352 nm, which are attributable to the (131) plane of the russellite phase Bi_2_WO_6_ and to the (101) plane of the anatase phase TiO_2_, respectively [40–41]. The SAED pattern of the 70%−Bi_2_WO_6_/TiO_2_-NT nanocomposite (Figure 4b) displays seven discernible diffraction rings which were denoted as 1−7. The rings 2, 4, 6, and 7 are attributed to the (004), (105), (204), and (215) planes of the anatase phase TiO_2_, while the rings 3 and 5 are assigned to the (202) and (262) planes of the russellite phase Bi_2_WO_6_, respectively [42–43]. Owing to the similar interplanar spacings of the (131) plane of the russellite phase Bi_2_WO_6_ and the (101) plane of the anatase phase TiO_2_, the thick ring 1 is ascribed to these two planes.

Figure 4c−h exhibit the EDX element mapping images of Bi, W, Ti, and O elements of the composite nanotube surface in the hierarchical 70%−Bi_2_WO_6_/TiO_2_-NT nanocomposite. The signals of Bi and W overlap to a great extent and uniformly distribute along the nanotube without aggregation. These signals are a little broader than those of Ti, revealing that Bi_2_WO_6_ nanoparticles spread along the TiO_2_ nanotube surface. Besides, the signals of Bi, W, and Ti interlaced among the composite nanotube, demonstrating the effective formation of heterostructures in between Bi_2_WO_6_ and TiO_2_ phases in the Bi_2_WO_6_/TiO_2_-NT nanocomposite.

**Figure 5:**
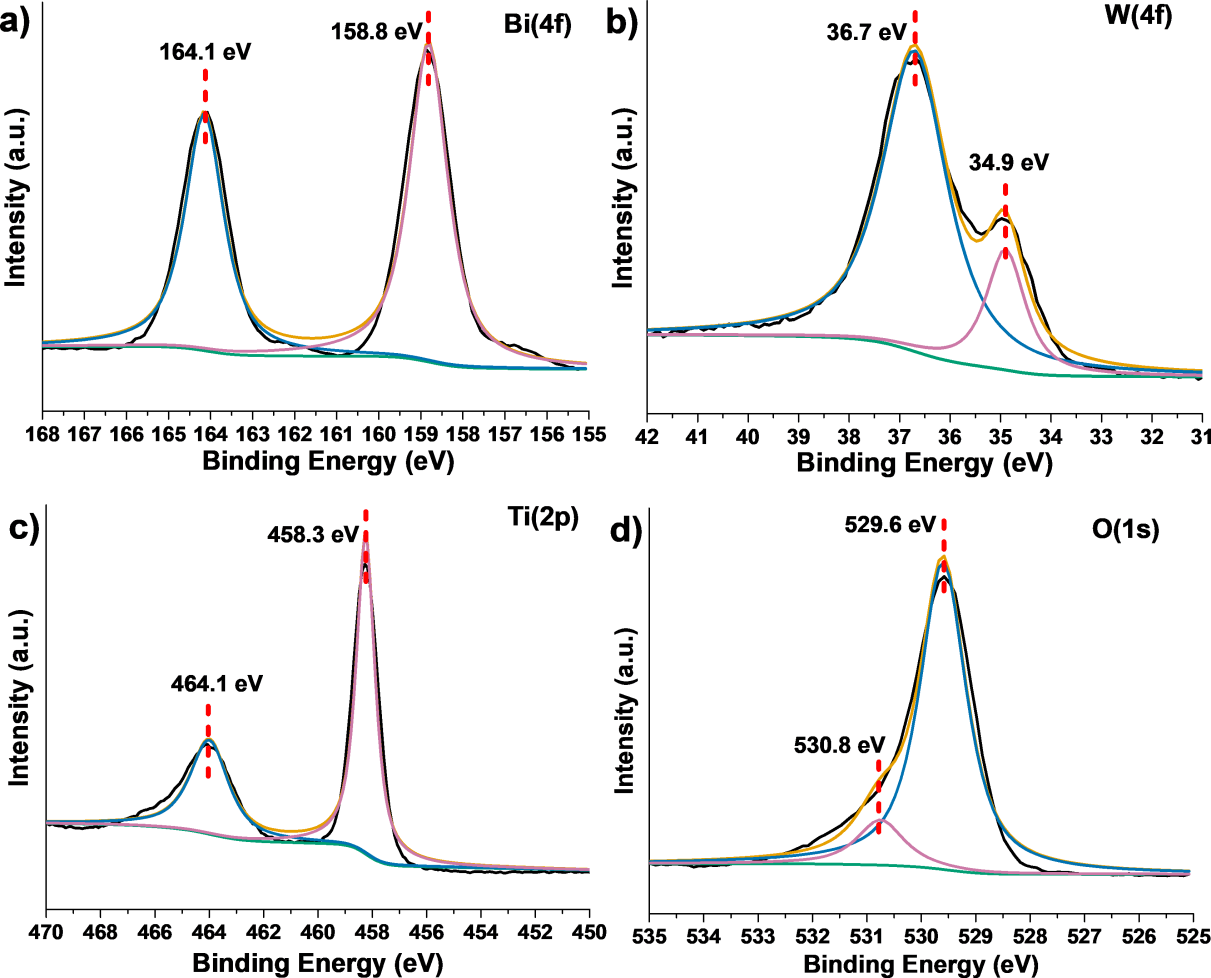
High-resolution XPS spectra of the (a) Bi 4f, (b) W 4f, (c) Ti 2p, and (d) O 1s regions of the hierarchical 70%−Bi_2_WO_6_/TiO_2_-NT nanocomposite.

As shown in Supporting Information File 1, Figure S3, the XPS survey spectrum of the 70%−Bi_2_WO_6_/TiO_2_-NT nanocomposite only displays the signals of Ti, O, Bi, and W. The high-resolution XPS spectrum of the Bi 4f region (Figure 5a) shows two peaks at 164.1 and 158.8 eV, which are indexed to the binding energies of Bi 4f_5/2_ and Bi 4f_7/2_, respectively, proving the existence of Bi(III) in Bi_2_WO_6_ [44]. The high-resolution XPS spectrum of the W 4f region (Figure 5b) represents the spin*–*orbit split lines of W 4f_5/2_ and W 4f_7/2_ at 36.7 and 34.9 eV, respectively, indicating the existence of W(VI) in Bi_2_WO_6_ [45]. As displayed in Figure 5c, there are two peaks at 464.1 and 458.3 eV that are attributed to the binding energies of Ti 2p_1/2_ and Ti 2p_3/2_ in the high-resolution XPS spectrum of the Ti 2p region [46], which show a distance of 5.8 eV. This demonstrates the Ti(IV) state in TiO_2_ in the 70%−Bi_2_WO_6_/TiO_2_-NT nanocomposite [47]. The high-resolution XPS spectrum of the O 1s region (Figure 5d) displays two peaks at 529.6 and 530.8 eV, which are ascribed to the lattice oxygen of the TiO_2_ and Bi_2_WO_6_ phases, as well as to the H_2_O molecules and –OH groups adsorbed on the sample surface [48–49]. As revealed by XPS, cellulose-derived Bi_2_WO_6_/TiO_2_-NT nanocomposites consist of Bi_2_WO_6_ and TiO_2_ phases, and this agrees well with the results of the aforementioned XRD, FTIR, HR-TEM, and SAED characterizations.

**Figure 6:**
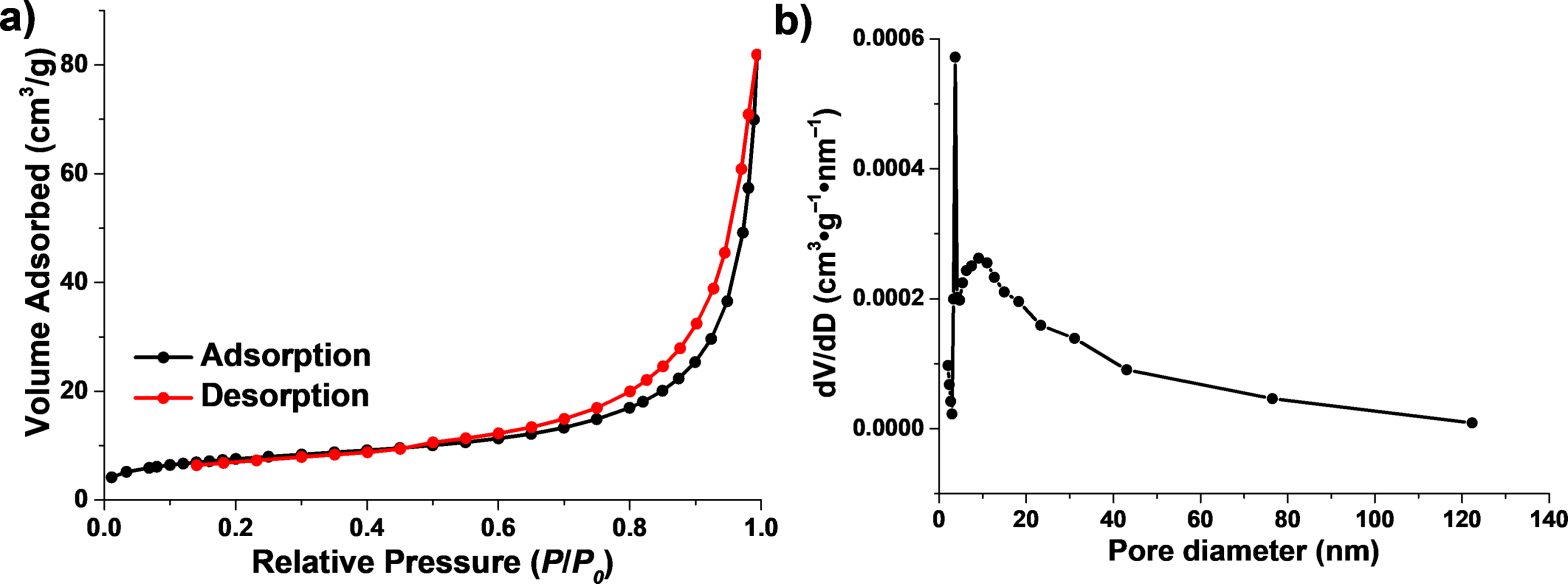
(a) N_2_ adsorption−desorption isotherms and (b) the pore size distribution pattern analyzed from the adsorption isotherm of the hierarchical 70%−Bi_2_WO_6_/TiO_2_-NT nanocomposite.

As shown in Figure 6a, based on the definition of IUPAC, the N_2_ adsorption−desorption isotherms of the 70%−Bi_2_WO_6_/TiO_2_-NT nanocomposite represent the type IV adsorption isotherm and the H3 type hysteresis in the range of 0.6 to 1.0 of the relative pressure (*P*/*P_0_*), demonstrating the mesoporous structure of the nanocomposite [50]. The specific surface area determined by the BET model (*S*_BET_) of the 70%−Bi_2_WO_6_/TiO_2_-NT nanocomposite is 26.3 m^2^·g^−1^, suggesting approximately the same value as that of pure TiO_2_-NT (26.4 m^2^·g^−1^) [51] but much higher than that of the pure Bi_2_WO_6_ powder (16.0 m^2^·g^−1^) [52]. This is mainly benefited from the uniform and compact dispersion of Bi_2_WO_6_ nanoparticles on the hierarchical TiO_2_ nanotubes without aggregation. The corresponding pore size distribution pattern analyzed by the BJH model exhibits a sharp peak at approx. 3 nm and a wide peak at approx. 10 nm, which are assigned to the mesopores of the TiO_2_ nanocrystallites in the TiO_2_ nanotubes and the gaps between the Bi_2_WO_6_ nanoparticles.

### Photocatalytic performance

**Figure 7:**
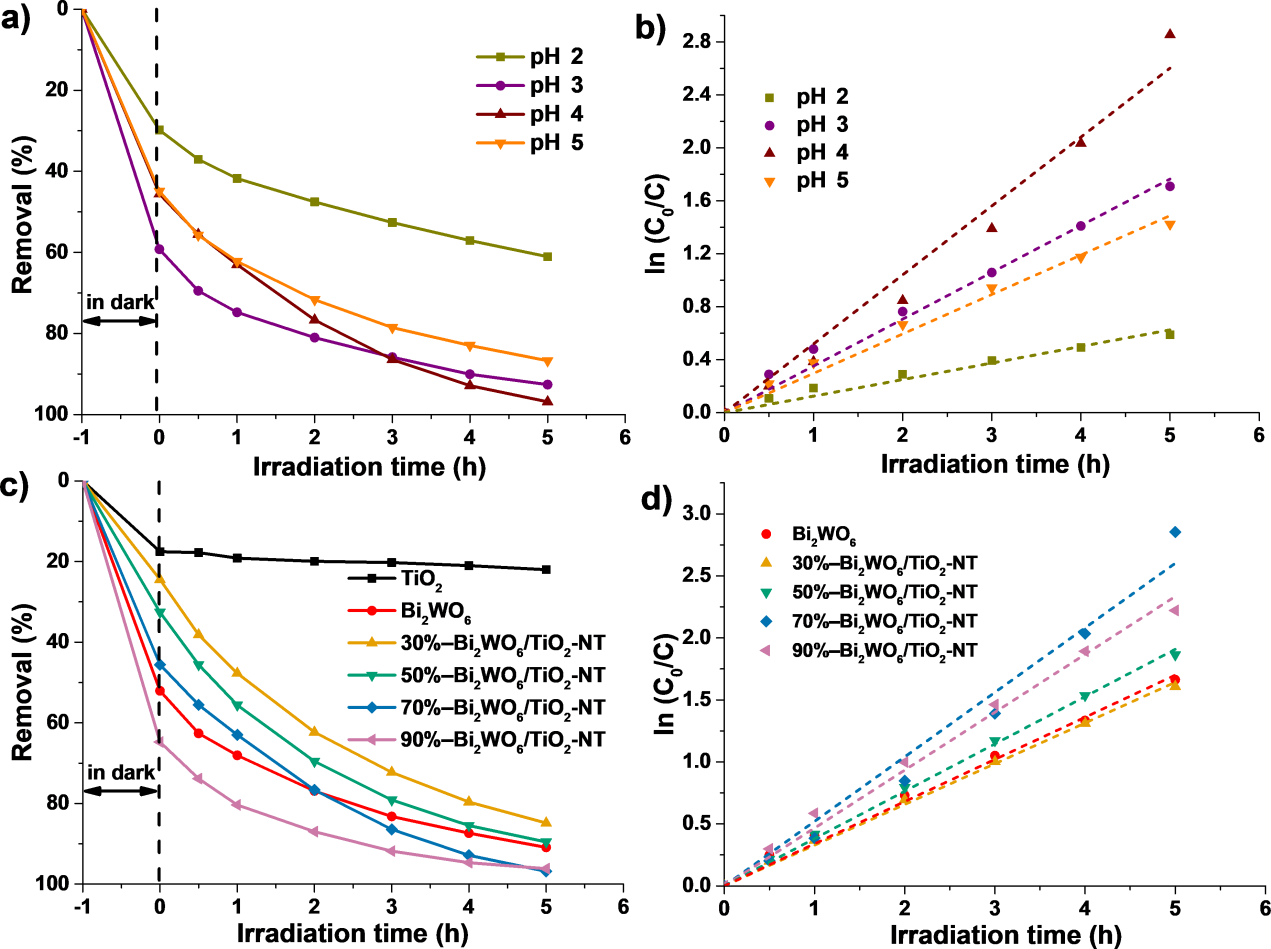
(a) Visible-light-induced (λ > 420 nm) photocatalytic reduction profiles toward the Cr(VI) pollutant solution (10 mg·L^−1^) under various pH conditions by the hierarchical 70%−Bi_2_WO_6_/TiO_2_-NT nanocomposite. (c) Visible-light-induced (λ > 420 nm) photocatalytic reduction profiles toward the Cr(VI) pollutant solution (10 mg·L^−1^, pH 4) by different samples, as well as (b,d) the corresponding linear fitting curves based on the pseudo-first-order kinetic model.

Cr(VI) and RhB pollutants are selected as the model pollutants for the evaluation of the photocatalytic performance of the hierarchical Bi_2_WO_6_/TiO_2_-NT nanocomposites. It is reported that the pH value of the Cr(VI) pollutant solution has an important influence in the photocatalytic reduction of Cr(VI) [53]. In order to investigate the photocatalytic reduction activities of the samples toward Cr(VI), the 70%−Bi_2_WO_6_/TiO_2_-NT nanocomposite was set as the representative photocatalyst for the exploration of optimal pH values of the Cr(VI) pollutant solution under visible-light irradiation. It was demonstrated that the reduction efficiency of Cr(VI) under the alkaline condition was poor because the newly formed Cr(OH)_3_ precipitates covered the active sites of the photocatalysts [54]. Hence, the photocatalytic reduction reactions of Cr(VI) were conducted under a series of acidic conditions. As revealed in Figure 7a and Figure 7b, the order of *K*_app_ values of the photocatalytic reactions under different pH conditions is pH 4 (0.52 h^−1^) > pH 3 (0.35 h^−1^) > pH 5 (0.30 h^−1^) > pH 2 (0.13 h^−1^), suggesting that the optimal pH value is pH 4. It has been proven that the pH condition of the Cr(VI) pollutant solution has great effects on the surface potentials of photocatalysts, and Cr(VI) ions exist in the form of HCrO_4_^−^ and Cr_2_O_7_^2−^ in the reactions shown in Equation 3 and Equation 4 [55].


[3] $$14{\text{H}}^{+}+{\text{Cr}}_{2}{\text{O}}_{7}{}^{2-}+6{\text{e}}^{-}\to 2{\text{Cr}}^{3+}+7{\text{H}}_{2}\text{O,}$$

[4] $$7{\text{H}}^{+}+{\text{HCrO}}_{4}{}^{-}+3{\text{e}}^{-}\to {\text{Cr}}^{3+}+4{\text{H}}_{2}\text{O.}$$

Under the pH 2 condition, the mass transfer efficiency of Cr(VI) is low due to the poor adsorption capacity of the photocatalyst toward HCrO_4_^−^ and Cr_2_O_7_^2−^. Although the concentration of H^+^ is high in the pollutant solution, the excessive adsorbed HCrO_4_^−^ and Cr_2_O_7_^2−^ block the active sites of the photocatalyst at pH 3, resulting in rather poor photocatalytic properties. In comparison with the pH 4 and pH 5 conditions, although the adsorption capacities toward HCrO_4_^−^ and Cr_2_O_7_^2−^ of the photocatalyst are similar, the photocatalytic reduction reaction of Cr(VI) is easier to take place at pH 4 owing to the higher concentration of H^+^ in the pollutant solution, leading to optimal photocatalytic performances. Similar results have been reported in the literature [54].

As shown in Figure 7c and Figure 7d, the photocatalytic performances of the samples toward the reduction of Cr(VI) were assessed under pH 4 and visible light (λ > 420 nm) conditions. In the dark adsorption stage, pure Bi_2_WO_6_ powder exhibits high adsorption capacity, while pure TiO_2_-NT presents low adsorption capacity toward the Cr(VI) pollutant, which is mainly due to the surface potentials of the related photocatalysts [56]. Hence, the adsorption capacities of the hierarchical Bi_2_WO_6_/TiO_2_-NT nanocomposites enhanced with the increase in Bi_2_WO_6_ contents in the corresponding nanocomposites.

When visible light is shone, the pure TiO_2_-NT displays little photocatalytic reduction activity toward Cr(VI), and the *K*_app_ values of the photocatalytic reactions of the other samples decrease in the following sequence: 70%−Bi_2_WO_6_/TiO_2_-NT (0.52 h^−1^) > 90%−Bi_2_WO_6_/TiO_2_-NT (0.47 h^−1^) > 50%−Bi_2_WO_6_/TiO_2_-NT (0.38 h^−1^) > pure Bi_2_WO_6_ powder (0.34 h^−1^) > 30%−Bi_2_WO_6_/TiO_2_-NT (0.33 h^−1^). It was concluded that all Bi_2_WO_6_/TiO_2_-NT nanocomposites present better photocatalytic performances toward the reduction of Cr(VI) except for the 30%−Bi_2_WO_6_/TiO_2_-NT nanocomposite. This is attributable to the cellulose-derived hierarchically interwoven structures as well as to the uniform and compact heterostructures formed in between the TiO_2_ and Bi_2_WO_6_ phases of the Bi_2_WO_6_/TiO_2_-NT nanocomposites, leading to higher separation and transfer efficiencies of the photoinduced electron−hole pairs. The optimal 70%−Bi_2_WO_6_/TiO_2_-NT photocatalyst achieves a removal percentage of 96.9% toward the reduction of Cr(VI) upon visible light irradiation for 5 h with a *K*_app_ value of 0.52 h^−1^, which is 1.5 folds higher than that of pure Bi_2_WO_6_ powder. Although the 70%−Bi_2_WO_6_/TiO_2_-NT nanocomposite does not have a superior adsorption capacity toward Cr(VI), the moderate density and uniform dispersion of Bi_2_WO_6_ nanoparticles on the hierarchical TiO_2_ nanotubes result in optimum migration efficiencies of photogenerated electrons and holes, which leads to the optimal photocatalytic reduction activity.

Similarly, as shown in Supporting Information File 1, Figure S4a, all Bi_2_WO_6_/TiO_2_-NT nanocomposites have larger adsorption capacities toward RhB than those of pure TiO_2_-NT and Bi_2_WO_6_ powder samples, except for the 90%−Bi_2_WO_6_/TiO_2_-NT nanocomposite, which is ascribed to the higher specific surface area as well as to close contact and uniform formation of the heterostructures in between the TiO_2_ and Bi_2_WO_6_ phases of the Bi_2_WO_6_/TiO_2_-NT nanocomposites. When visible light (λ > 420 nm) is irradiated, as shown in Supporting Information File 1, Figure S4, the *K*_app_ values of all samples decrease as follows: 70%−Bi_2_WO_6_/TiO_2_-NT (0.65 h^−1^) > 50%−Bi_2_WO_6_/TiO_2_-NT (0.45 h^−1^) > 30%−Bi_2_WO_6_/TiO_2_-NT (0.36 h^−1^) > 90%−Bi_2_WO_6_/TiO_2_- NT (0.14 h^−1^) > pure Bi_2_WO_6_ powder (0.09 h^−1^) > pure TiO_2_-NT (0.05 h^−1^). The optimal 70%−Bi_2_WO_6_/TiO_2_-NT nanocomposite reveals a degradation percentage of 98.2% under visible-light irradiation for 6 h with a *K*_app_ value of 0.65 h^−1^, which is 13.0 and 7.2 times as high as those of pure TiO_2_-NT and Bi_2_WO_6_ powder samples, respectively. As a comparison, all hierarchical Bi_2_WO_6_/TiO_2_-NT nanocomposites present better photocatalytic properties than those of pure TiO_2_-NT and Bi_2_WO_6_ powder samples, which is attributed to the three-dimensional porous network structures inherited from the original cellulose configuration and the formation of compact heterostructures in between the TiO_2_ and Bi_2_WO_6_ phases of the Bi_2_WO_6_/TiO_2_-NT nanocomposites. This ultimately leads to enhanced adsorption capacities toward RhB as well as to rapid transfer and separation of the photogenerated electron−hole pairs.

As shown in Supporting Information File 1, Figure S5a and S5c, self-reduction of Cr(VI) and self-degradation of RhB during the photocatalytic processes are negligible, and the adsorption of the optimal 70%−Bi_2_WO_6_/TiO_2_-NT nanocomposite toward Cr(VI) and RhB stopped after the achievement of adsorption−desorption equilibrium, suggesting that the photocatalytic reactions of the hierarchical Bi_2_WO_6_/TiO_2_-NT nanocomposites are generated from intrinsic reactions. As a comparison, the *K*_app_ values of the 70%−Bi_2_WO_6_/TiO_2_-NT nanocomposite (0.52 h^−1^) toward the reduction of Cr(VI) are, respectively, 1.9 and 2.4 times higher than those of the Bi_2_WO_6_/TiO_2_ sample prepared without cellulose template (0.28 h^−1^) and the Bi_2_WO_6_-TiO_2_ sample prepared by physical blending (0.22 h^−1^) ( Supporting Information File 1, Figure S5a,b). The *K*_app_ values of the 70%−Bi_2_WO_6_/TiO_2_-NT nanocomposite (0.65 h^−1^) toward the reduction of Cr(VI) (Supporting Information File 1, Figure S5c and d) are, respectively, 3.6 and 8.1 times higher than those of the Bi_2_WO_6_/TiO_2_ (0.18 h^−1^) and Bi_2_WO_6_-TiO_2_ (0.08 h^−1^) samples. This result reveals that the cellulose-derived three-dimensional network structure promotes the homogeneous dispersion of Bi_2_WO_6_ nanoparticles on TiO_2_ nanotubes, as well as an intense interaction and uniform formation of heterostructures in between the TiO_2_ and Bi_2_WO_6_ phases of the Bi_2_WO_6_/TiO_2_-NT nanocomposites, resulting in the superior photocatalytic performances.

As shown in Table 1, in comparison with other Bi_2_WO_6_/TiO_2_ composites reported in the literature, cellulose-derived Bi_2_WO_6_/TiO_2_-NT nanocomposites deliver higher *K*_app_ values and larger increments as compared to pure TiO_2_ under more rigorous conditions of visible-light (λ > 420 nm) irradiation and lower dosage of photocatalyst toward the reduction of Cr(VI) or degradation of RhB. The excellent photocatalytic activities of hierarchical Bi_2_WO_6_/TiO_2_-NT nanocomposites are benefited from the uniform deposition of Bi_2_WO_6_ nanoparticles on TiO_2_ nanotubes and from compact heterostructures built in between the TiO_2_ and Bi_2_WO_6_ phases, which is due to the three-dimensional interwoven structures that duplicated from the natural cellulose template.

**Table 1:** Comparison of visible-light-induced (λ > 420 nm) photocatalytic performances toward the degradation of RhB or reduction of Cr(VI) with other reported Bi_2_WO_6_/TiO_2_ composites in the literature.

Bi_2_WO_6_/TiO_2_ composites	Light source	Catalyst dosage	Concentration and volume of pollutants	*K*_app_ (h^−1^)	Increment compared with pure TiO_2_	Ref.

Bi_2_WO_6_/TiO_2_-NT	350 W Xe, λ > 420 nm	10 mg	RhB: 10 mg L^−1^, 20 mL	0.65	13.0	this work
			Cr(VI): 10 mg·L^−1^, 20 mL	0.52	–	
Sb^3+^ doped Bi_2_WO_6_/TiO_2_	Xe lamp with AM 1.5 filter	–	RhB: 9.58 mg·L^−1^	0.55	–	[48]
Bi_2_WO_6_/TiO_2_/Pt	Xe lamp 320 nm < λ < 780 nm	100 mg	RhB: 20 mg·L^−1^, 100 mL	1.26	7.0	[57]
Bi_2_WO_6_/TiO_2_ nanotubes	400 W Xe, λ > 420 nm	200 mg	RhB: 50 mg·L^−1^, 220 mL	0.66	8.7	[58]
Bi_2_WO_6_/mesoporous TiO_2_ nanotubes	300 W Xe with UV cut-off filter	50 mg	Cr(VI): 20 mg·L^−1^, 100 mL	0.33	–	[59]

As shown in Supporting Information File 1, Table S3, in comparison with cellulose-derived Ag_2_O-nanoparticle/TiO_2_-nanotube (Ag_2_O-NP/TiO_2_-NT) composites [31], g-C_3_N_4_/TiO_2_- nanotube (g-C_3_N_4_/TiO_2_-NT) composites [32], and H_3_PW_12_O_40_/TiO_2_ nanocomposites [33] reported by our group, the hierarchical Bi_2_WO_6_/TiO_2_-NT composite delivered similar three-dimensional interwoven structures that comprised the composite nanotubes. The Ag_2_O-NP/TiO_2_-NT and H_3_PW_12_O_40_/TiO_2_ composites exhibited excellent photocatalytic performances under UV light irradiation, while the g-C_3_N_4_/TiO_2_-NT and Bi_2_WO_6_/TiO_2_-NT composites had a wider light response to the visible spectral region. Besides, under visible light irradiation, Bi_2_WO_6_/TiO_2_-NT composites show better photocatalytic degradation activities than that of g-C_3_N_4_/TiO_2_-NT composites. Based on these cellulose-derived nanocomposites, structure–activity relationships between photocatalytic activities and structures containing the three-dimensional hierarchical network of the natural cellulose template and the compositions of the composite photocatalysts are revealed.

**Figure 8:**
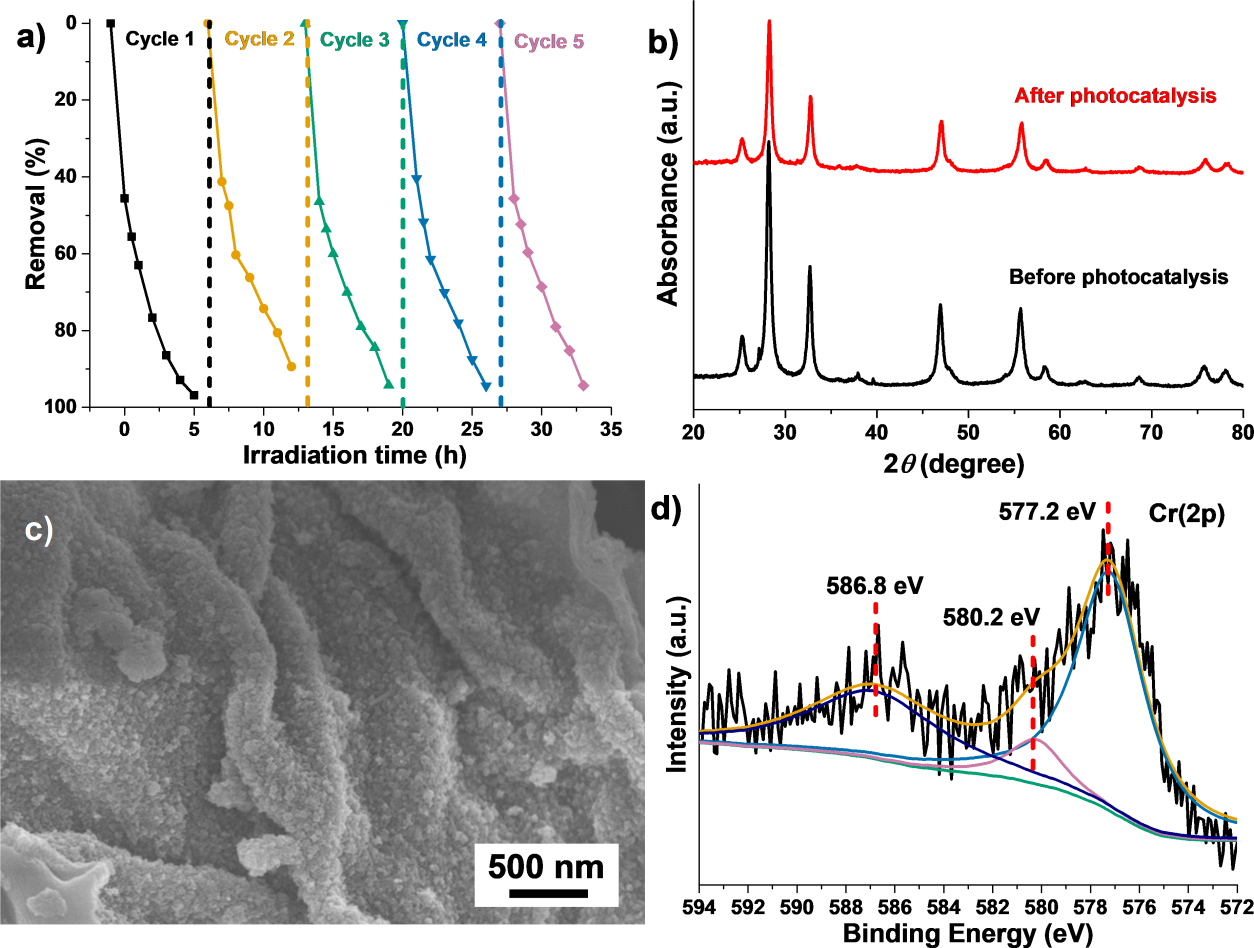
(a) The visible-light-induced (λ > 420 nm) photocatalytic reduction profiles toward the Cr (VI) pollutant solution (10 mg·L^−1^, pH 4) for five cycles by the hierarchical 70%−Bi_2_WO_6_/TiO_2_-NT nanocomposite. (b) XRD patterns of the 70%−Bi_2_WO_6_/TiO_2_-NT nanocomposites before and after five-cycle photocatalysis. (c) The FE-SEM image and (d) high-resolution XPS spectrum of the Cr 2p region of the 70%−Bi_2_WO_6_/TiO_2_-NT nanocomposite after five-cycle photocatalysis.

To evaluate the photocatalytic stability of cellulose-derived Bi_2_WO_6_/TiO_2_-NT nanocomposites, the 70%−Bi_2_WO_6_/TiO_2_-NT nanocomposite was chosen as the representative photocatalyst toward the reduction of Cr(VI) and degradation of RhB. As exhibited in Figure 8a, the percentage removal on the fifth cycle only decreased 2% as compared with the first cycle of the 70%−Bi_2_WO_6_/TiO_2_-NT photocatalyst toward the reduction of Cr(VI) under visible light (λ > 420 nm). This demonstrates the rather high photocatalytic stability of Bi_2_WO_6_/TiO_2_-NT nanocomposites on the reduction of Cr(VI). The XRD patterns of the 70%−Bi_2_WO_6_/TiO_2_-NT nanocomposites before and after the photocatalytic reactions (Figure 8b) exhibit a high degree of consistency, suggesting that the crystal structure of the sample is maintained during the cyclic photocatalysis. As shown in Figure 8c, FE-SEM images of the 70%−Bi_2_WO_6_/TiO_2_-NT nanocomposite after photocatalysis still present the cellulose-derived three-dimensionally porous network structure, and the Bi_2_WO_6_ nanoparticles are still uniformly and tightly coated on the TiO_2_ nanotube surfaces, revealing the morphological and structural stabilities of the Bi_2_WO_6_/TiO_2_-NT nanocomposite. In the high-resolution XPS spectrum of the Cr 2p region of the 70%−Bi_2_WO_6_/TiO_2_-NT nanocomposite after photocatalysis (Figure 8d), there are two strong peaks at 586.8 and 577.2 eV that were attributed to the binding energies of Cr 2p_1/2_ and Cr 2p_3/2_, which are assigned to Cr(III) [60]. Besides, the weak peak at 580.2 eV is indexed to the spin–orbit split line of Cr 2p_3/2_, which corresponds to Cr(VI) [54], suggesting that the 70%−Bi_2_WO_6_/TiO_2_-NT nanocomposite effectively reduced Cr(VI) into Cr(III) in five cycles. Benefiting from the cellulose-derived hierarchical network structures together with the crystal and morphological/structural stabilities of the Bi_2_WO_6_/TiO_2_-NT nanocomposite, the adsorbed Cr species on the nanocomposite have no effect on the cyclic photocatalysis, proving the photocatalytic stability of the Bi_2_WO_6_/TiO_2_-NT nanocomposite.

Analogously, as shown in Supporting Information File 1, Figure S6a, the degradation percentage only declines to 13% after five-cycle photocatalytic reactions toward the degradation of RhB, which is mainly due to the loss of the powder photocatalyst during the centrifugation procedure in the cycling processes. The XRD pattern (Supporting Information File 1, Figure S6b) and FE-SEM images (Supporting Information File 1 Figure S6c and Figure S6d) of the 70%−Bi_2_WO_6_/TiO_2_-NT nanocomposite after photocatalysis reveal high stabilities of the crystal and morphological structures. It is concluded that the stabilities of the crystal and morphological structures of the Bi_2_WO_6_/TiO_2_-NT nanocomposite result in the superior photocatalytic stability toward the degradation of RhB.

### Photocatalytic mechanism

**Figure 9:**
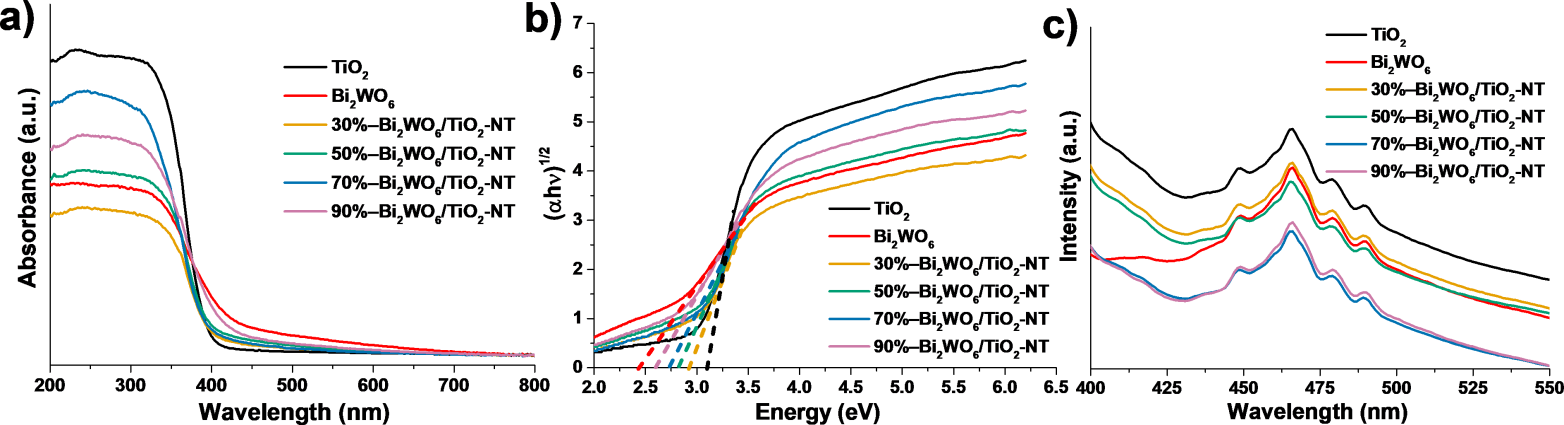
(a) UV–vis DRS, (b) bandgaps determined by the intercept on the *x*-axis of the respective Tauc plots, and (c) PL emission spectra under the excitation at 360 nm of the pure TiO_2_-NT sample, pure Bi_2_WO_6_ powder sample, and the hierarchical Bi_2_WO_6_/TiO_2_-NT nanocomposites.

As presented in Figure 9a, all the UV–vis DRS of the hierarchical Bi_2_WO_6_/TiO_2_-NT nanocomposites show the regions of UV light response and visible light response. The UV–vis DRS of the pure TiO_2_-NT sample shows an absorption edge at approx. 400 nm that is ascribed to the UV light response without the visible light response, while the UV–vis DRS of the pure Bi_2_WO_6_ powder sample displays an absorption edge at approx. 505 nm, which corresponds to both UV and visible light responses. As compared with the pure TiO_2_-NT sample, the absorption edges in the UV–vis DRS of the Bi_2_WO_6_/TiO_2_-NT nanocomposites extend to approx. 425, 440, 455, and 490 nm, suggesting that the visible light responses of the nanocomposites are remarkably strengthened and the response enhances with the increased Bi_2_WO_6_ content in the respective nanocomposite.

As shown in Figure 9b, the order of the bandgaps of the samples is as follows: pure Bi_2_WO_6_ powder (2.40 eV) < 90%−Bi_2_WO_6_/TiO_2_-NT (2.55 eV) < 70%−Bi_2_WO_6_/TiO_2_-NT (2.73 eV) < 50%−Bi_2_WO_6_/ TiO_2_-NT (2.83 eV) < 30%−Bi_2_WO_6_/TiO_2_-NT (2.92 eV) < pure TiO_2_-NT (3.12 eV). This reveals that the bandgaps of the Bi_2_WO_6_/TiO_2_-NT nanocomposites decrease with the increase in the Bi_2_WO_6_ content in the corresponding nanocomposites. As compared with the pure TiO_2_-NT sample, the enhanced visible light responses and decreased bandgaps of the Bi_2_WO_6_/TiO_2_-NT nanocomposites are attributed to the wider visible-light-responsive region of the Bi_2_WO_6_ component and the uniform heterostructures built in between the TiO_2_ and Bi_2_WO_6_ phases in the nanocomposites, leading to the production of more carriers when induced by visible light. It is reported that the change of binding energy due to the atomic bonding or charge transfer transition of the conduction bands in between TiO_2_ and Bi_2_WO_6_ phases in the Bi_2_WO_6_/TiO_2_-NT nanocomposites results in the aforementioned enhancement [39].

The PL emission spectra of the related samples under the excitation at 360 nm are presented in Figure 9c, which are applied to evaluate the separation and transfer efficiencies of the samples. All PL spectra exhibit a strong peak at 456 nm and other three weak peaks at 448, 479, and 490 nm, which are indexed to the recombination of photoinduced electron−hole pairs, freely excited electrons, surface defects, and oxygen vacancies on the band edges, respectively [36,61]. It is apparent that the PL intensities of the Bi_2_WO_6_/TiO_2_-NT nanocomposites at 465 nm are all weaker than those of pure TiO_2_-NT and Bi_2_WO_6_ powder samples, except for the 30%−Bi_2_WO_6_/TiO_2_-NT nanocomposite. This demonstrates that the deposition of Bi_2_WO_6_ nanoparticles on the TiO_2_ nanotubes is effective to inhibit the recombination of photogenerated electron−hole pairs.

In comparison with the Bi_2_WO_6_/TiO_2_-NT nanocomposites with varied contents of the Bi_2_WO_6_ component, the weakest PL intensity of the 70%−Bi_2_WO_6_/TiO_2_-NT nanocomposite demonstrates its highest separation and transfer efficiencies of photogenerated electron−hole pairs, which is advantageous to the photocatalytic reduction of Cr(VI) and degradation of RhB. Although the 90%−Bi_2_WO_6_/TiO_2_-NT nanocomposite has the largest amount of heterostructures in between the TiO_2_ and Bi_2_WO_6_ phases owing to its highest content of the Bi_2_WO_6_ component, the PL intensity is a little higher than that of the 70%−Bi_2_WO_6_/TiO_2_-NT nanocomposite, which is due to the fact that excessive Bi_2_WO_6_ nanoparticles act as the recombination centers and inhibit the transfer of the photoinduced electrons and holes. However, due to the lower contents of the Bi_2_WO_6_ component, the heterostructures in the 30%−Bi_2_WO_6_/TiO_2_-NT and 50%−Bi_2_WO_6_/TiO_2_-NT nanocomposites are less than that of the 70%−Bi_2_WO_6_/ TiO_2_-NT nanocomposite, causing the lower separation and transfer efficiencies of the photogenerated electron−hole pairs.

In comparison with the Bi_2_WO_6_/TiO_2_ sample prepared without the cellulose template and the Bi_2_WO_6_-TiO_2_ sample prepared by physical blending (Supporting Information File 1, Figure S7), the cellulose-derived 70%−Bi_2_WO_6_/TiO_2_-NT nanocomposite possesses wider absorption edge in the UV–vis DRS, corresponding narrower bandgap, and weaker PL intensity in the PL spectra, suggesting stronger response to visible light and more efficient separation of the photogenerated electron−hole pairs.

**Figure 10:**
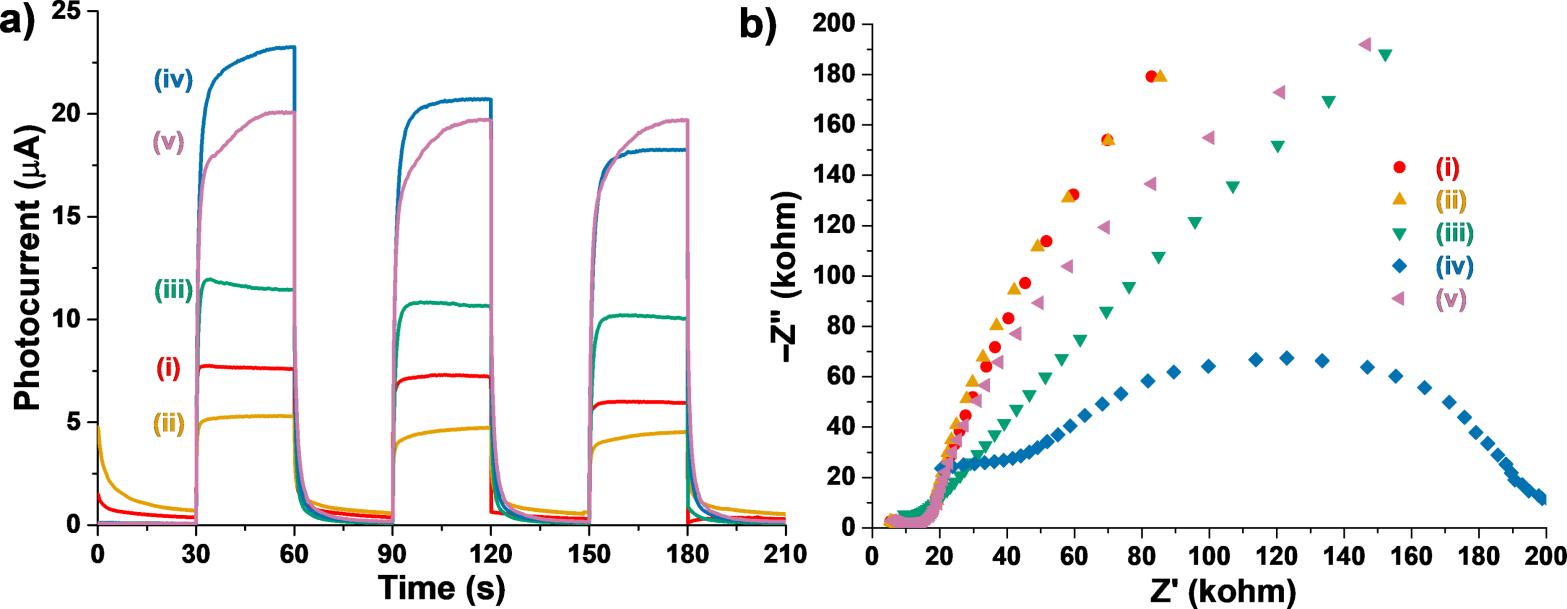
(a) The transient photocurrent responses and (b) EIS Nyquist plots of (i) pure Bi_2_WO_6_ samples and hierarchical (ii) 30%−Bi_2_WO_6_/TiO_2_-NT, (iii) 50%−Bi_2_WO_6_/TiO_2_-NT, (iv) 70%−Bi_2_WO_6_/TiO_2_-NT, and (v) 90%−Bi_2_WO_6_/TiO_2_-NT nanocomposites.

It is reported that the transient photocurrent responses of the samples depend on the amounts of photogenerated charges and the kinetics of charge separation of the corresponding electrodes under irradiation of light [62]. As shown in Figure 10a, under irradiation of visible light (λ > 420 nm) at intervals of 30 s, all the Bi_2_WO_6_/TiO_2_-NT nanocomposites exhibit higher photocurrent responses than that of the pure Bi_2_WO_6_ powder sample except for the 30%−Bi_2_WO_6_/TiO_2_-NT nanocomposite, revealing that the recombination of photoinduced electrons and holes is inhibited on the interface of the Bi_2_WO_6_/TiO_2_-NT heterostructures, which promotes the generation of more effective photogenerated charges. Besides, the 70%−Bi_2_WO_6_/TiO_2_-NT and 90%−Bi_2_WO_6_/TiO_2_-NT nanocomposites behave similarly but have higher photocurrent responses than those of the 50%−Bi_2_WO_6_/TiO_2_-NT nanocomposite, which performs analog results as compared to the PL characterization, suggesting the most effective separation and transfer of photogenerated carriers of the 70%−Bi_2_WO_6_/TiO_2_-NT and 90%−Bi_2_WO_6_/TiO_2_-NT nanocomposites.

EIS Nyquist plots of samples are usually employed to determine the transfer resistances and efficiencies of the photogenerated charges of the corresponding electrodes. As displayed in Figure 10b, the circular arc radii in the EIS Nyquist plots of all the Bi_2_WO_6_/TiO_2_-NT nanocomposites are smaller than those of the pure Bi_2_WO_6_ powder sample apart from the 30%−Bi_2_WO_6_/TiO_2_-NT nanocomposite. This demonstrates more effective transfer of photogenerated charges of the Bi_2_WO_6_/TiO_2_-NT nanocomposites, which is benefited from the uniform and compact heterostructures built in the Bi_2_WO_6_/TiO_2_-NT nanocomposites. The smallest circular arc radius in the EIS Nyquist plots of the 70%−Bi_2_WO_6_/TiO_2_-NT nanocomposite proves that it has the highest transfer efficiencies of the photoinduced carriers among the samples.

As compared with the Bi_2_WO_6_/TiO_2_ sample prepared without the cellulose template and the Bi_2_WO_6_-TiO_2_ sample prepared by physical blending (Supporting Information File 1, Figure S8), the cellulose-derived 70%−Bi_2_WO_6_/TiO_2_-NT nanocomposite represents higher photocurrent responses and smaller circular arc radius in the EIS Nyquist plots, demonstrating more effective separation and shift of the photogenerated carriers, which promotes the generation of more effective charges under visible-light irradiation.

As revealed in the structural characterizations, the Bi_2_WO_6_/TiO_2_ sample prepared without the cellulose template shows several aggregated Bi_2_WO_6_ particles on the surface of bulk TiO_2_. Conversely, the cellulose-derived three-dimensionally interwoven structures of the Bi_2_WO_6_/TiO_2_-NT nanocomposites promote the homogeneous distribution of the Bi_2_WO_6_ nanoparticles on the TiO_2_ nanotubes, the compact interaction in between the two phases, as well as the uniform formation of the heterostructures. The integrated characterization results of the aforementioned UV–vis DRS, PL spectra, transient photocurrent responses, and EIS Nyquist plots reveal that these factors facilitate stronger responses upon visible light and more effective separation of the photogenerated electron−hole pairs of the Bi_2_WO_6_/TiO_2_-NT nanocomposites, leading to the production of more effective carriers for photocatalytic reactions. This contributes to the superior photocatalytic activities toward the reduction of Cr(VI) and degradation of RhB.

**Figure 11:**
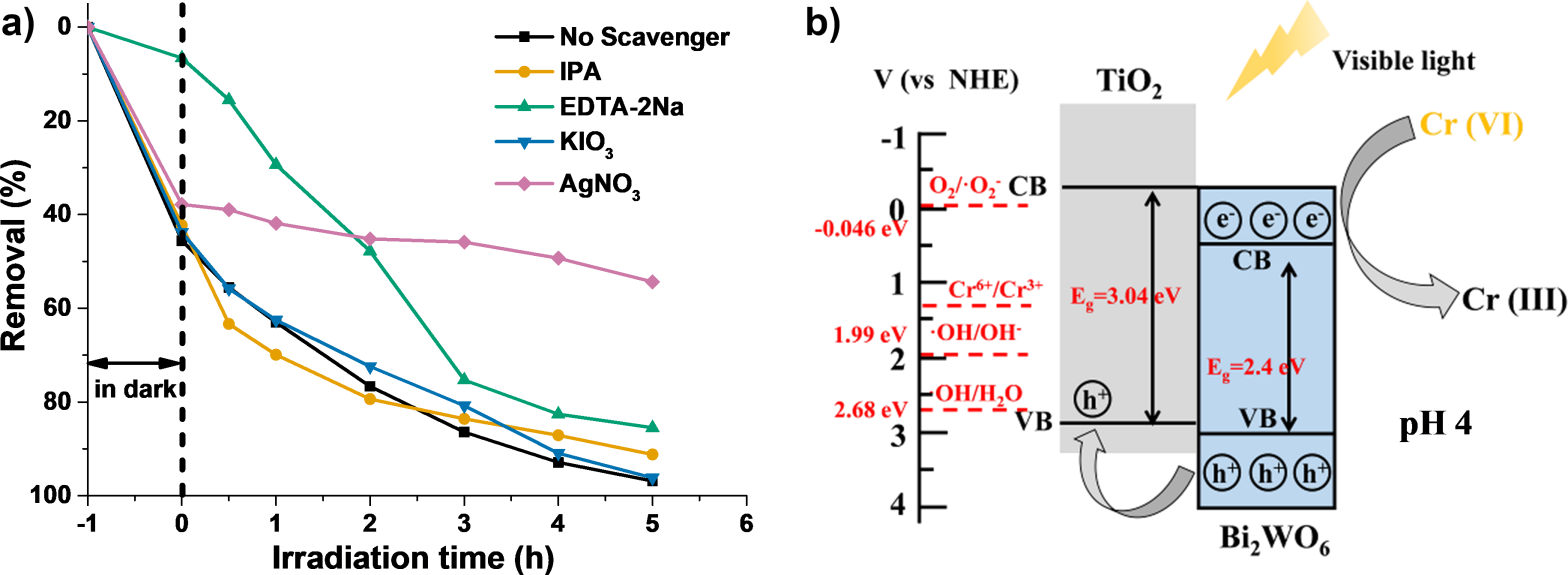
(a) Visible-light- induced (λ > 420 nm) photocatalytic reduction profiles toward the Cr(VI) pollutant solution (10 mg·L^−1^, pH 4) where IPA, EDTA-2Na, KIO_3_, and AgNO_3_ were added. The hierarchical 70%−Bi_2_WO_6_/TiO_2_-NT nanocomposite was used as the photocatalyst. (b) Schematic illustration of the photocatalytic reduction mechanism toward the Cr(VI) pollutant (pH 4) by the hierarchical Bi_2_WO_6_/TiO_2_-NT nanocomposite under visible light (λ > 420 nm) irradiation.

As shown in Figure 11a, IPA (0.1 M), EDTA-2Na (10.0 mM), KIO_3_ (0.1 M), and AgNO_3_ (0.1 M) were added into the Cr(VI) pollutant solution for the capture of ^•^OH, h^+^, ^•^O_2_^−^ and e^−^ species, respectively, with the optimal 70%−Bi_2_WO_6_/TiO_2_-NT nanocomposite as the photocatalyst. It is apparent that only the addition of AgNO_3_ suppresses the photocatalytic reduction of Cr(VI), suggesting that e^−^ is the uppermost species present during photocatalysis.

The edge potentials of the valence band (*E*_VB_) and conduction band (*E*_CB_) of the TiO_2_ and Bi_2_WO_6_ semiconductors are determined according to the Equation 5 and Equation 6 [31]:


[5] $$E_{\text{VB}}=\chi -E^{\text{e}}+0.5E_{\text{g}},$$

[6] $$E_{\text{CB}}=E_{\text{VB}}-E_{\text{g}},$$

where χ, *E*^e^, and *E*_g_ represent the geometric mean of the absolute electronegativity of the atoms in the semiconductors, the free electron energy on the hydrogen scale (approx. 4.5 eV), and the bandgaps of semiconductors based on the UV–vis DRS characterizations, respectively. It is reported that the χ values of the TiO_2_ and Bi_2_WO_6_ semiconductors are 5.81 and 6.21 eV, respectively [52]; while the *E*_g_ values of the TiO_2_ and Bi_2_WO_6_ semiconductors are determined to be 3.12 and 2.40 eV, respectively. Therefore, the *E*_VB_ and *E*_CB_ values of the TiO_2_ semiconductor are calculated to be 2.87 and −0.25 eV, and those of the Bi_2_WO_6_ semiconductor are 2.91 and 0.51 eV, respectively.

Based on the results of the above photoelectrochemical characterizations and the scavenger experiments of the active species, the photocatalytic mechanism toward the reduction of Cr(VI) by the cellulose-derived Bi_2_WO_6_/TiO_2_-NT nanocomposites is illustrated in Figure 11b. When the Bi_2_WO_6_/TiO_2_-NT nanocomposite is irradiated by visible light (λ > 420 nm), the electrons and holes are only generated on the CB and VB of the Bi_2_WO_6_ phase. Due to the compact and uniform heterostructures formed in between the TiO_2_ and Bi_2_WO_6_ phases and the suitable band positions of the two phases, holes on the VB of the Bi_2_WO_6_ phase are rapidly transferred to that of the TiO_2_ phase, while electrons are still left on the CB of the Bi_2_WO_6_ phase. This leads to the separation of the photogenerated electron−hole pairs on the Bi_2_WO_6_ phase. Because of the higher standard redox potential of Cr(VI)/Cr(III) (1.36 eV vs NHE) [63] and lower standard redox potential of O_2_/^•^O_2_^−^ (−0.046 eV vs NHE) than those of the *E*_CB_ of the Bi_2_WO_6_ phase, the electrons on the CB of the Bi_2_WO_6_ phase directly transform Cr(VI) into Cr(III) rather than reduce O_2_ into ^•^O_2_^−^ species. Although the holes on the VBs of the TiO_2_ and Bi_2_WO_6_ phases are able to ideally oxidize H_2_O molecules and OH^−^ into ^•^OH species, they actually have little effect on the photocatalytic reduction of Cr(VI) according to the aforementioned capture experiment of the active species.

The primary active species and the possible mechanism toward the photocatalytic degradation of RhB by the hierarchical Bi_2_WO_6_/TiO_2_-NT nanocomposites are explored. As shown in Supporting Information File 1, Figure S9a, the addition of IPA (0.1 M) or EDTA-2Na (0.1 M) has great effects on the photodegradation of RhB, while the addition of p-BQ (5.0 mM) makes no difference, revealing that ^•^OH and h^+^ species are the dominating active species during the photocatalytic degradation of RhB by the 70%−Bi_2_WO_6_/TiO_2_-NT nanocomposite. As illustrated in Supporting Information File 1, Figure S9b, holes on the VB of the Bi_2_WO_6_ phase are shifted to that of the TiO_2_ phase rapidly after the irradiation of visible light, leading to the efficient separation of photogenerated electron−hole pairs. The electrons left on the CB of the Bi_2_WO_6_ phase are not able to react with O_2_ molecules to yield ^•^O_2_^−^ species due to the lower standard redox potential of O_2_/^•^O_2_^−^ (−0.046 eV vs NHE) than the *E*_CB_ of the Bi_2_WO_6_ phase. Owing to the higher *E*_VB_ of the TiO_2_ and Bi_2_WO_6_ phases than to the standard redox potential of ^•^OH/H_2_O (2.68 eV vs NHE) and ^•^OH/OH^−^ (1.99 eV vs NHE), a part of the holes on the VBs of the TiO_2_ and Bi_2_WO_6_ phases oxidizes H_2_O and OH^−^ into ^•^OH species, which decompose RhB together with another part of the unreacted holes, and the holes play a greater role than the ^•^OH species.

## Conclusion

In conclusion, a series of hierarchically porous network structures of Bi_2_WO_6_/TiO_2_-NT nanocomposites were fabricated by depositing Bi_2_WO_6_ nanoparticles with various densities on the cellulose-derived three-dimensional TiO_2_ nanotube surfaces via a solvothermal process. As compared with pure TiO_2_-NT, pure Bi_2_WO_6_ powder, the Bi_2_WO_6_/TiO_2_ sample prepared without the cellulose template, and the Bi_2_WO_6_-TiO_2_ sample prepared by physical blending, Bi_2_WO_6_/TiO_2_-NT nanocomposites had superior photocatalytic performances toward the reduction of Cr(VI) and degradation of RhB under visible-light irradiation (λ > 420 nm). It is demonstrated that the promoted photocatalytic activities of the Bi_2_WO_6_/TiO_2_-NT nanocomposites are benefited from the three-dimensionally interwoven structures inherited from the initial cellulose template and the uniform and compact heterostructures formed in between the TiO_2_ and Bi_2_WO_6_ phases, which are mainly ascribed to the unique structure of the cellulose template. The current research reveals the structure–activity relationships of the cellulose-derived nanocomposite, providing an insight for the preparation of photocatalytic composite materials that combines the unique structures of specific natural substances with excellent properties of related guest components for the treatment of pollutants.

## Supporting Information

Dosages of Bi(NO_3_)_3_·5H_2_O and Na_2_WO_4_·2H_2_O reagents in the preparation processes. EDX spectra and components of the related samples. Electron micrographs of the control samples. XPS survey spectrum of the 70%−Bi_2_WO_6_/TiO_2_-NT nanocomposite. The visible-light-induced photocatalytic degradation profiles and the corresponding linear fitting curves toward the photodegradation of RhB pollutant solution by the samples. The reduction profiles toward the Cr(VI) pollutant solution and the degradation profiles toward the RhB pollutant solution as well as the corresponding linear fitting curves. The visible-light-induced photocatalytic degradation profiles toward the photodegradation of the RhB pollutant solution for five cycles by the 70%−Bi_2_WO_6_/TiO_2_-NT nanocomposite, and the XRD patterns and FE-SEM images of the sample after 5-cycle photocatalysis. The UV–vis DRS, bandgaps and PL emission spectra of the related samples. The transient photocurrent responses and EIS Nyquist plots of the control samples. The visible-light-induced photocatalytic degradation profiles toward the RhB pollutant solution added with IPA, EDTA-2Na, and p-BQ by the 70%−Bi_2_WO_6_/TiO_2_-NT nanocomposite, and the schematic illustration of the corresponding photocatalytic degradation mechanism. The comparison of the hierarchical Bi_2_WO_6_/TiO_2_-nanotube composite with other cellulose-derived nanocomposites that were reported by our group.

File 1 Additional figures.

## References

[1] Bolisetty S, Peydayesh M, Mezzenga R (2019). Chem Soc Rev.

[2] Sang Y, Cao X, Dai G, Wang L, Peng Y, Geng B (2020). J Hazard Mater.

[3] Ou B, Wang J, Wu Y, Zhao S, Wang Z (2020). Chem Eng J.

[4] Patnaik S, Sahoo D P, Parida K M (2020). J Colloid Interface Sci.

[5] Kong M, Li Y, Chen X, Tian T, Fang P, Zheng F, Zhao X (2011). J Am Chem Soc.

[6] Yang Y, Wu S, Li Y, Zhang Q, Zhao X (2020). J Mater Chem A.

[7] Zeng M, Li Y, Mao M, Bai J, Ren L, Zhao X (2015). ACS Catal.

[8] Zeng G, You H, Du M, Zhang Y, Ding Y, Xu C, Liu B, Chen B, Pan X (2021). Chem Eng J.

[9] Zhang Y, Sun A, Xiong M, Macharia D K, Liu J, Chen Z, Li M, Zhang L (2021). Chem Eng J.

[10] Ariga K, Mori T, Li J (2019). Langmuir.

[11] Ariga K (2021). Molecules.

[12] Ariga K, Shionoya M (2021). Bull Chem Soc Jpn.

[13] Ariga K (2020). Chem Sci.

[14] Ariga K (2020). Adv Inorg Chem.

[15] Ariga K (2020). Beilstein J Nanotechnol.

[16] Chen G, Sciortino F, Ariga K (2021). Adv Mater Interfaces.

[17] Ariga K, Vinu A, Yamauchi Y, Ji Q, Hill J P (2012). Bull Chem Soc Jpn.

[18] Ariga K, Ishihara S, Abe H (2016). CrystEngComm.

[19] Abe H, Liu J, Ariga K (2016). Mater Today.

[20] Du Z, Cheng C, Tan L, Lan J, Jiang S, Zhao L, Guo R (2018). Appl Surf Sci.

[21] Zhang N, Ciriminna R, Pagliaro M, Xu Y-J (2014). Chem Soc Rev.

[22] Colón G, Murcia-López S, Hidalgo M C, Navío J A (2010). Chem Commun.

[23] Murcia-López S, Vaiano V, Sannino D, Hidalgo M C, Navío J A (2015). Res Chem Intermed.

[24] Murcia-López S, Villa K, Andreu T, Morante J R (2014). ACS Catal.

[25] Sun X, Zhang H, Wei J, Yu Q, Yang P, Zhang F (2016). Mater Sci Semicond Process.

[26] Chen C, Tian W, Xu W, Cao F, Li L (2019). ChemElectroChem.

[27] Sun F, Qi H, Xie Y, Ma Q, He W, Xu D, Wang G, Yu W, Wang T, Dong X (2020). J Alloys Compd.

[28] Lv C, Lan X, Wang L, Wang C, Liu X, Shi J (2020). ChemistrySelect.

[29] Lin Z, Huang J (2019). Dalton Trans.

[30] Lin Z, Li S, Huang J (2021). Chem – Asian J.

[31] Lin Z, Lu Y, Huang J (2019). Cellulose.

[32] Lin Z, Yu B, Huang J (2020). Langmuir.

[33] Lin Z, Huang J (2021). Sep Purif Technol.

[34] Sharma S, Ibhadon A O, Francesconi M G, Mehta S K, Elumalai S, Kansal S K, Umar A, Baskoutas S (2020). Nanomaterials.

[35] Zhang M (2020). J Mater Sci: Mater Electron.

[36] Wang R, Xu M, Xie J, Ye S, Song X (2020). Colloids Surf, A.

[37] Wang H, Wang L, Ye S, Song X (2019). Food Hydrocolloids.

[38] Chai Y h, Zhou F, Zhu Z (2019). Chem Phys Lett.

[39] Ji L, Liu B, Qian Y, Yang Q, Gao P (2020). Adv Powder Technol.

[40] Wang K, Wei W, Lou Z, Zhang H, Wang L (2019). Appl Surf Sci.

[41] Shang M, Wang W, Zhang L, Sun S, Wang L, Zhou L (2009). J Phys Chem C.

[42] Zhang Y, Fei L, Jiang X, Pan C, Wang Y (2011). J Am Ceram Soc.

[43] Luo Y, Liu X, Huang J (2013). CrystEngComm.

[44] Fang G, Liu J, Wu J, Li M, Yan X, Wang D (2019). Appl Surf Sci.

[45] Chen G, Wang Y, Shen Q, Xiong X, Ren S, Dai G, Wu C (2020). Ceram Int.

[46] Guo Q, Huang Y, Xu H, Luo D, Huang F, Gu L, Wei Y, Zhao H, Fan L, Wu J (2018). Solid State Sci.

[47] Shi H, Yu Y, Zhang Y, Feng X, Zhao X, Tan H, Khan S U, Li Y, Wang E (2018). Appl Catal, B.

[48] Wang Q, Li H, Yu X, Jia Y, Chang Y, Gao S (2020). Electrochim Acta.

[49] Ghoreishian S M, Ranjith K S, Lee H, Ju H-i, Zeinali Nikoo S, Han Y-K, Huh Y S (2020). J Hazard Mater.

[50] Yang C, Huang Y, Li F, Li T (2016). J Mater Sci.

[51] Liu X, Gu Y, Huang J (2010). Chem – Eur J.

[52] Fang G, Li M, Shen H, Yang S, Israr J (2021). Mater Sci Semicond Process.

[53] Cherifi Y, Barras A, Addad A, Ouddane B, Roussel P, Chaouchi A, Szunerits S, Boukherroub R (2021). Chemosphere.

[54] Zhang G, Chen D, Li N, Xu Q, Li H, He J, Lu J (2019). Appl Catal, B.

[55] Li Y-X, Wang X, Wang C-C, Fu H, Liu Y, Wang P, Zhao C (2020). J Hazard Mater.

[56] Li X, Chen D, Li N, Xu Q, Li H, He J, Lu J (2020). J Hazard Mater.

[57] Lu Y, Zhao K, Zhao Y, Zhu S, Yuan X, Huo M, Zhang Y, Qiu Y (2015). Colloids Surf, A.

[58] Ma F-Y, Yang Y, Li N, Yang Q-L, Li S-J, Shen L-Y (2017). Chin J Inorg Chem.

[59] Cheng L, Liu S, He G, Hu Y (2020). RSC Adv.

[60] Guo T, Yang S, Chen Y, Yang L, Sun Y, Shang Q (2021). Environ Sci Pollut Res.

[61] S R, Thomas J (2017). J Environ Chem Eng.

[62] Arif M, Zhang M, Yao J, Yin H, Li P, Hussain I, Liu X (2019). J Alloys Compd.

[63] Song X-Y, Chen Q-L (2019). J Nanopart Res.

