# Supplementary material for: Hierarchical Bi2WO6/TiO2-nanotube composites derived from natural cellulose for visible-light photocatalytic treatment of pollutants
Source: Beilstein J Nanotechnol. 2022 Aug 4;13:745–62. doi: 10.3762/bjnano.13.66 (PMC9359189; doi:10.3762/bjnano.13.66)
Supplement: File 1 — Additional figures. [file Beilstein_J_Nanotechnol-13-745-s001.pdf]

BEILSTEIN JOURNAL OF NANOTECHNOLOGY

Supporting Information

for

Hierarchical Bi_2WO_6/TiO_2-nanotube composites derived from natural cellulose for visible-light photocatalytic treatment of pollutants

Zehao Lin, Zhan Yang and Jianguo Huang

Beilstein J. Nanotechnol. **2022,** *13,* 745–762. doi:10.3762/bjnano.13.66

Additional figures

License and Terms: This is a supporting information file under the terms of the Creative Commons Attribution License (https://creativecommons.org/licenses/by/4.0). Please note that the reuse, redistribution and reproduction in particular requires that the author(s) and source are credited and that individual graphics may be subject to special legal provisions.
The license is subject to the *Beilstein Journal of Nanotechnology* terms and conditions: (https://www.beilstein-journals.org/bjnano/terms)

Table S1: The dosages of $Bi(NO_3)_3 \cdot 5H_2O$ and $Na_2WO_4 \cdot 2H_2O$ reagents in the preparation processes of the hierarchical Bi_2WO_6/TiO_2-NT nanocomposites.

Nanocomposites	$Bi(NO_3)_3 \cdot 5H_2O$/mg	$Na_2WO_4 \cdot 2H_2O$/mg
30%−Bi_2WO_6/TiO_2-NT	23.8	8.1
50%−Bi_2WO_6/TiO_2-NT	55.6	18.9
70%−Bi_2WO_6/TiO_2-NT	129.7	44.1
90%−Bi_2WO_6/TiO_2-NT	500.3	170.2

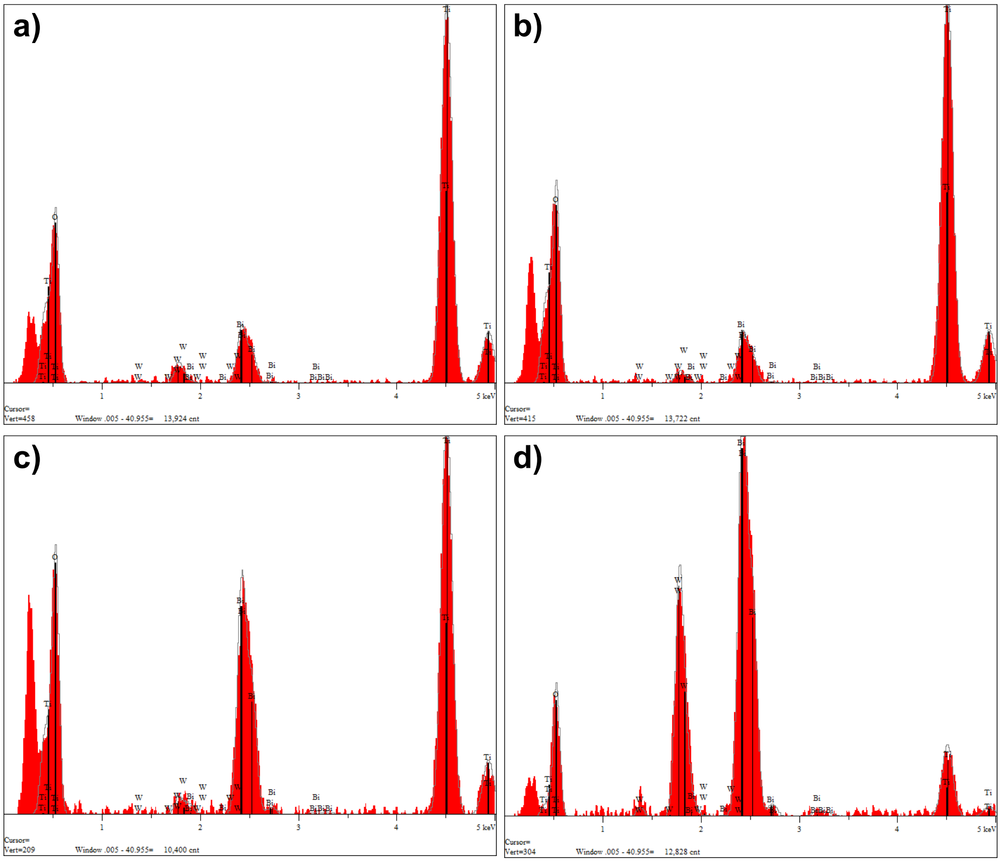

Figure S1: EDX spectra of the hierarchical (a) 30%−Bi_2WO_6/TiO_2-NT, (b) 50%−Bi_2WO_6/TiO_2-NT, (c) 70%−Bi_2WO_6/TiO_2-NT, and (d) 90%−Bi_2WO_6/TiO_2-NT nanocomposites.

Table S2: Practical mass contents of Ti and Bi elements, as well as the Bi_2WO_6 component in the hierarchical Bi_2WO_6/TiO_2-NT nanocomposites.

Nanocomposites	Practical mass content of Ti/wt %	Practical mass content of Bi/wt %	Practical mass content of Bi_2WO_6/wt %
30%−Bi_2WO_6/TiO_2-NT	47.04	29.26	38.4
50%−Bi_2WO_6/TiO_2-NT	37.56	44.52	54.3
70%−Bi_2WO_6/TiO_2-NT	22.40	60.29	72.9
90%−Bi_2WO_6/TiO_2-NT	3.79	74.50	95.2

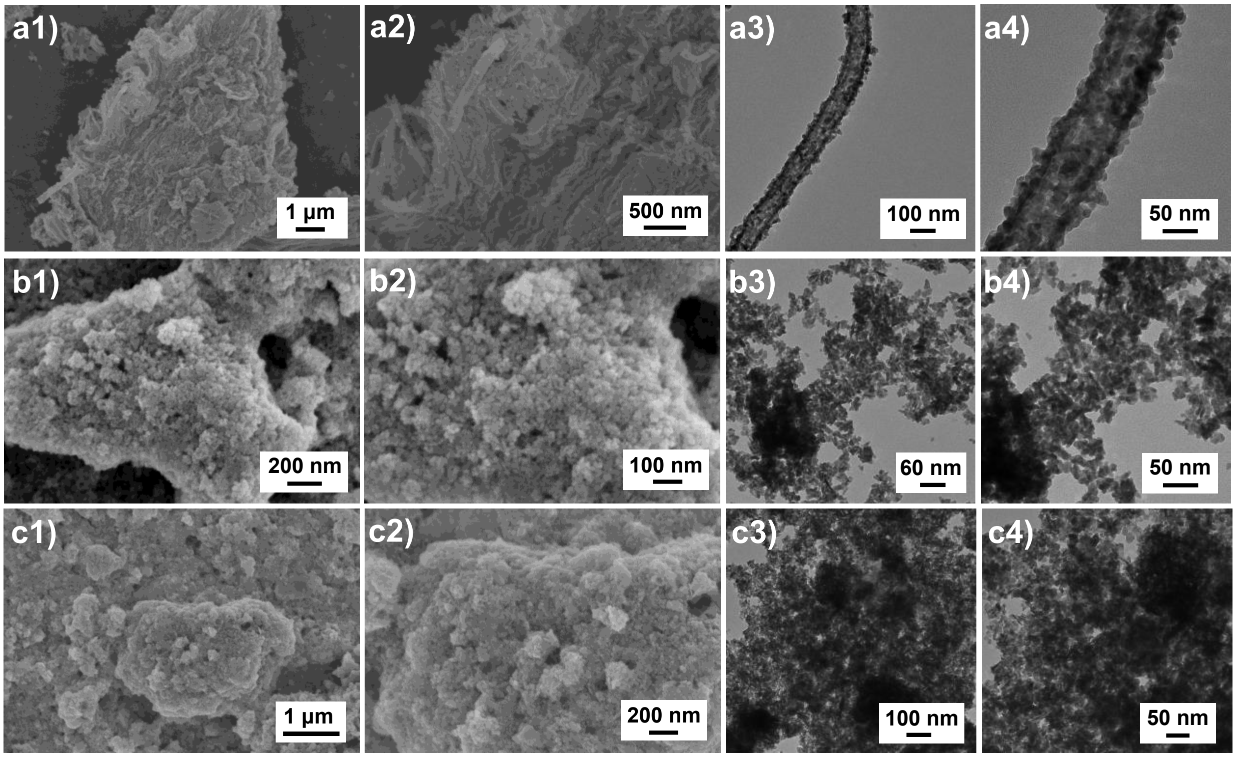

Figure S2: Electron micrographs of the (a1−a4) pure TiO_2-NT sample, (b1−b4) pure Bi_2WO_6 powder sample, and (c1−c4) the Bi_2WO_6/TiO_2 sample prepared without the cellulose template. The first two columns represent the FE-SEM images, and the last two columns exhibit the TEM images of the corresponding samples.

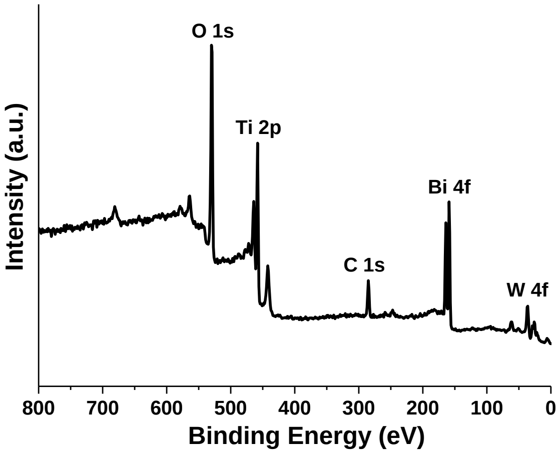

Figure S3: XPS survey spectrum of the hierarchical 70%−Bi_2WO_6/TiO_2-NT nanocomposite.

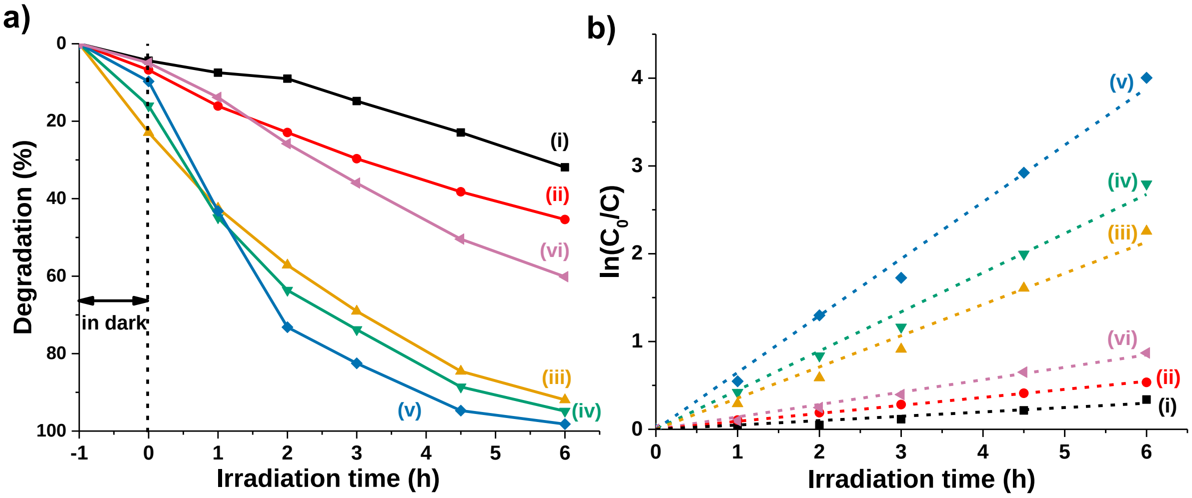

Figure S4: (a) The visible-light (λ > 420 nm) induced photocatalytic degradation profiles and (b) the corresponding linear fitting curves based on the pseudo-first-order kinetic model towards the photodegradation of RhB pollutant solution (10 mg·L^{-1}) by the (i) pure TiO_2-NT,

(ii) pure Bi_2WO_6 powder, as well as the hierarchical (iii) 30%−Bi_2WO_6/TiO_2-NT, (iv) 50%−Bi_2WO_6/TiO_2-NT, (v) 70%−Bi_2WO_6/TiO_2-NT, and (vi) 90%−Bi_2WO_6/TiO_2-NT nanocomposites.

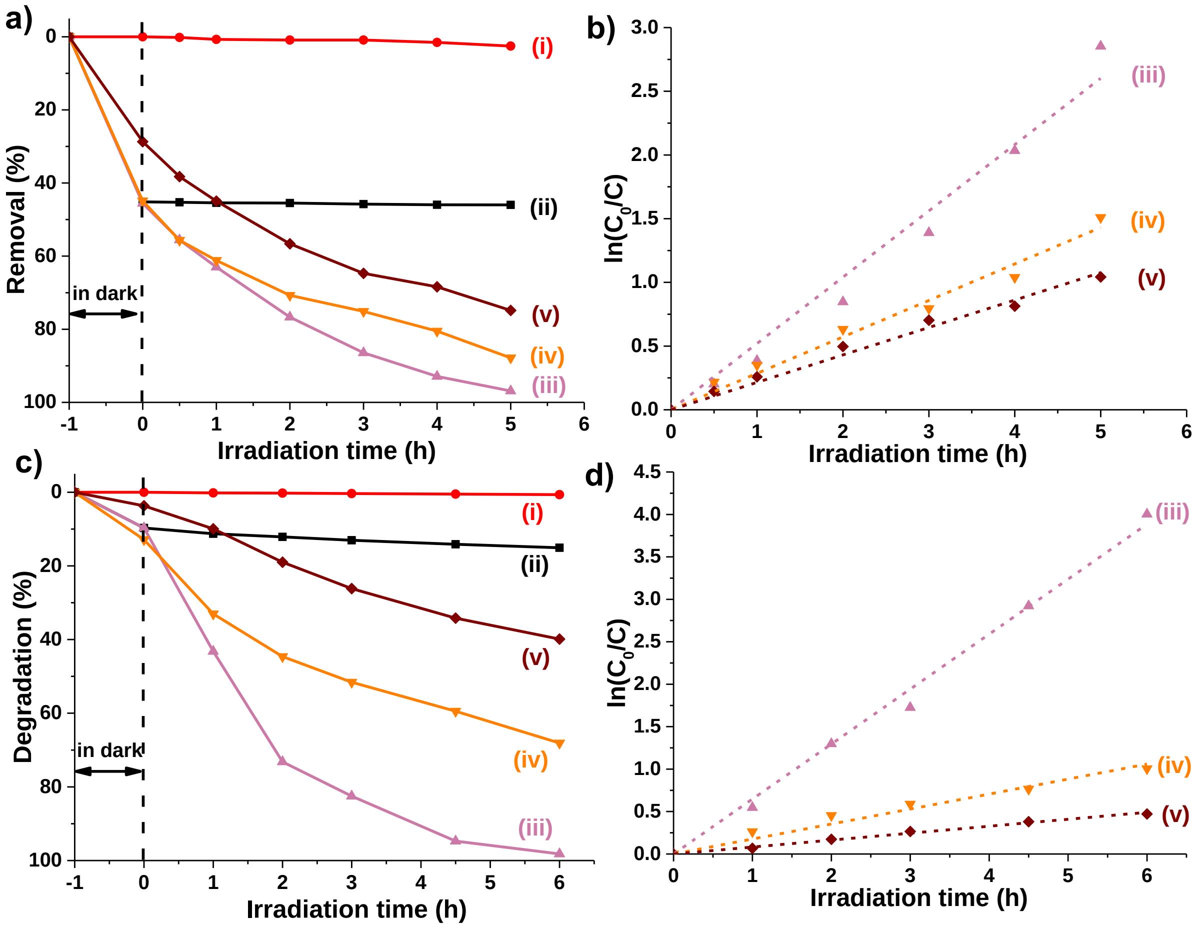

Figure S5: (a) The reduction profiles towards Cr(VI) pollutant solution (10 mg·L^{-1}, pH 4) and (c) the degradation profiles towards RhB pollutant solution (10 mg·L^{-1}) as well as (b,d) the corresponding linear fitting curves based on the pseudo-first-order kinetic model under different conditions. (i) The self-reduction reaction of Cr(VI) or self-degradation reaction of RhB without photocatalysts under visible-light (λ > 420 nm) irradiation. (ii) The self-adsorption by the hierarchical 70%−Bi_2WO_6/TiO_2-NT nanocomposite towards Cr(VI) or RhB pollutant solution. And the visible-light (λ > 420 nm) induced photocatalytic reactions towards Cr(VI) or RhB pollutant solution by employing (iii) the hierarchical 70%−Bi_2WO_6/TiO_2-NT

nanocomposite, (iv) the Bi_2WO_6/TiO_2 sample prepared without the cellulose template, and (v) the Bi_2WO_6-TiO_2 sample prepared by physical blend as the photocatalysts.

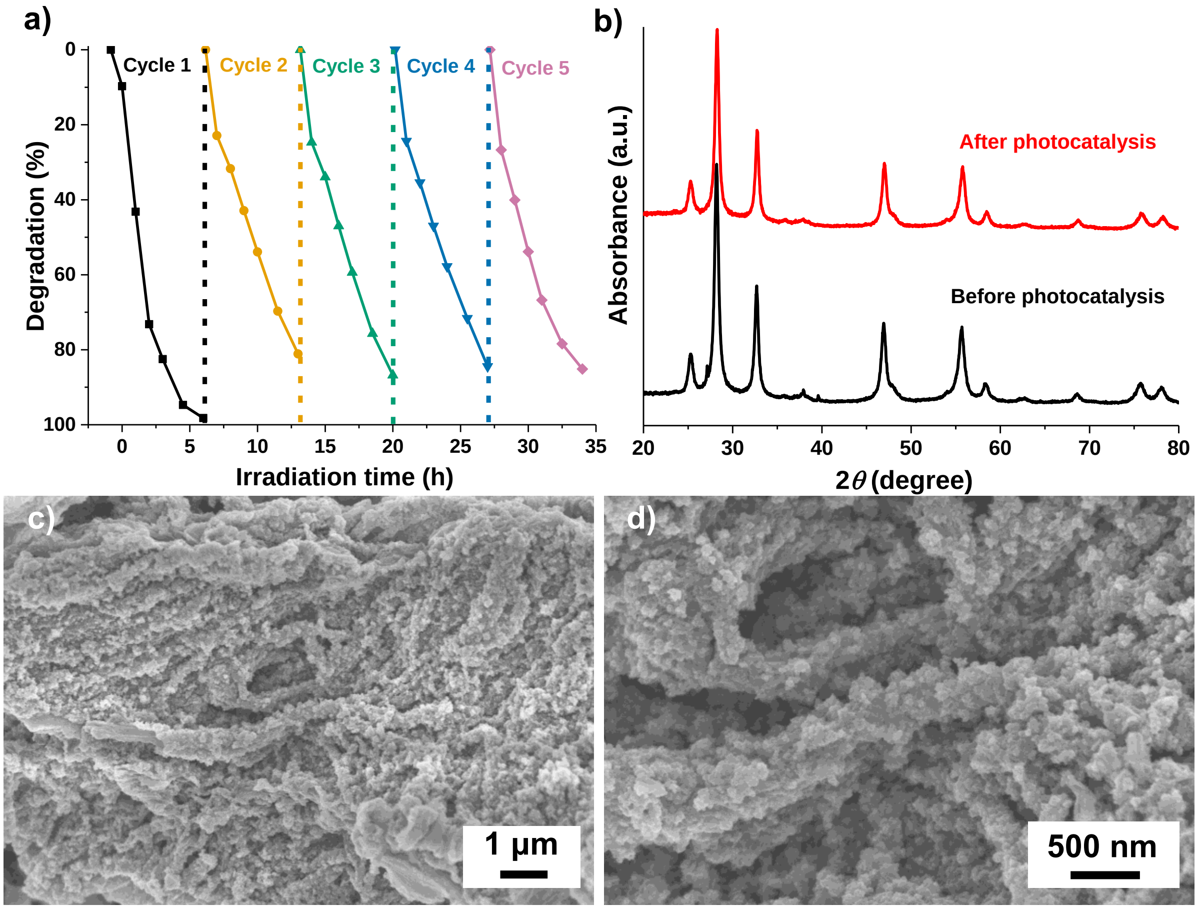

Figure S6: (a) The visible-light (λ > 420 nm) induced photocatalytic degradation profiles towards the photodegradation of RhB pollutant solution (10 mg·L^{-1}) for five cycles by the hierarchical 70%−Bi_2WO_6/TiO_2-NT nanocomposite. (b) The XRD patterns of the 70%−Bi_2WO_6/TiO_2-NT nanocomposites before and after 5-cycle photocatalysis. (c,d) The FE-SEM images of the 70%−Bi_2WO_6/TiO_2-NT nanocomposite after 5-cycle photocatalysis.

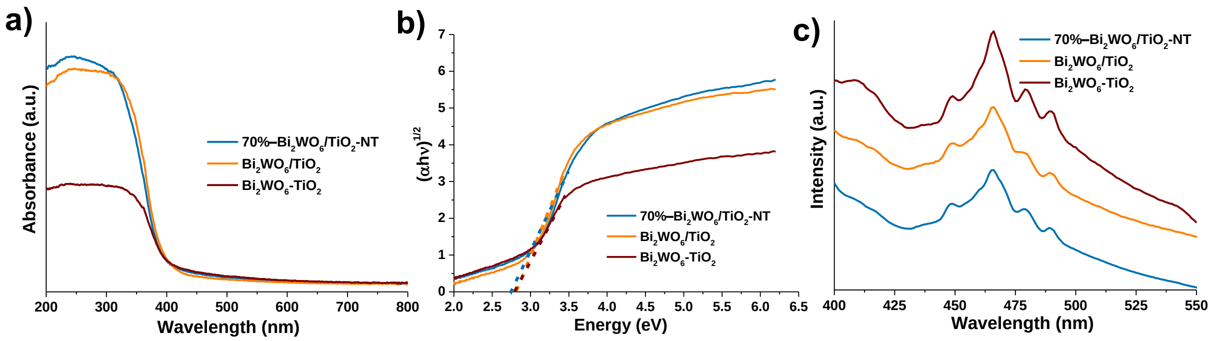

Figure S7: (a) The UV–vis DRS, (b) the band gaps determined by the intercept on the *x*-axis of the respective Tauc plots, and the PL emission spectra under the excitation of 360 nm of the hierarchical 70%−Bi_2WO_6/TiO_2-NT nanocomposite, the Bi_2WO_6/TiO_2 sample prepared without the cellulose template, and the Bi_2WO_6-TiO_2 sample prepared by physical blend.

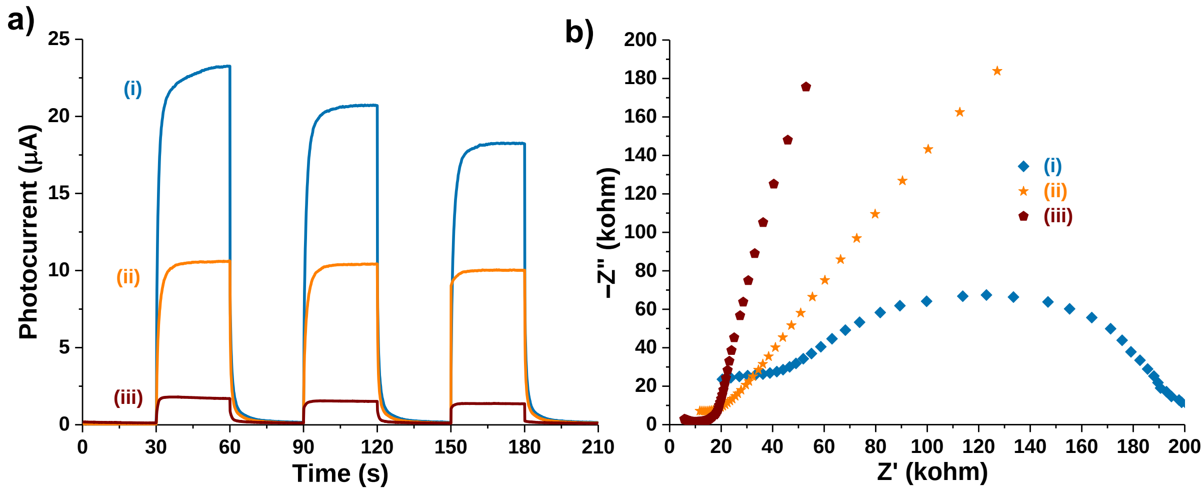

Figure S8: (a) The transient photocurrent responses and (b) EIS Nyquist plots of (i) the hierarchical 70%−Bi_2WO_6/TiO_2-NT nanocomposite, (ii) the Bi_2WO_6/TiO_2 sample prepared without the cellulose template, and (iii) the Bi_2WO_6-TiO_2 sample prepared by physical blend.

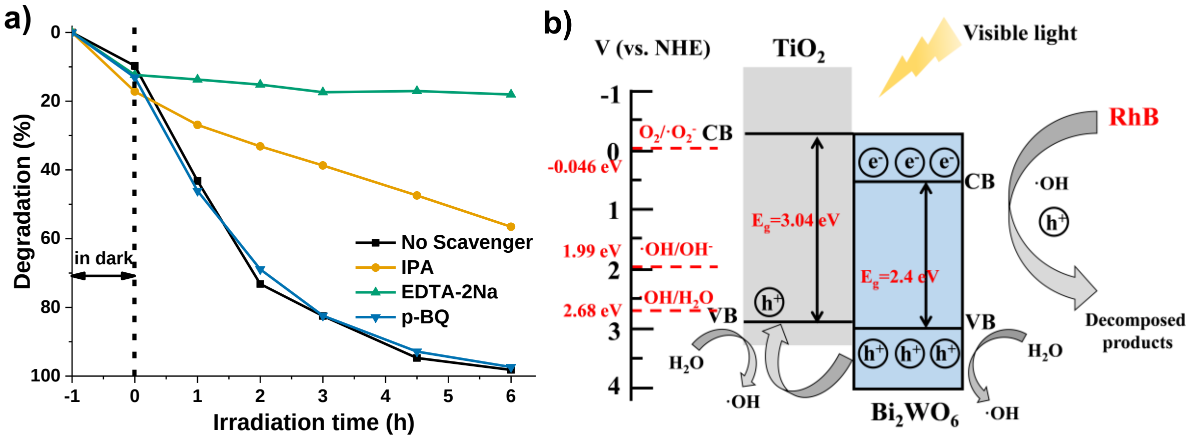

Figure S9: (a) The visible-light (λ > 420 nm) induced photocatalytic degradation profiles towards the RhB pollutant solution (10 mg·L^{-1}) added with IPA, EDTA-2Na, and p-BQ by the hierarchical 70%−Bi_2WO_6/TiO_2-NT nanocomposite. (b) The schematic illustration of the photocatalytic degradation mechanism towards the RhB pollutant by the hierarchical Bi_2WO_6/TiO_2-NT nanocomposite under the irradiation of visible light (λ > 420 nm).

Table S3: Comparison of the hierarchical Bi_2WO_6/TiO_2-nanotube composite with the natural cellulose substance derived Ag_2O-nanoparticle/TiO_2-nanotube composite, g-C_3N_4/TiO_2-nanotube composite, and $H_3PW_{12}O_{40}/TiO_2$ nanocomposite reported by our group.

composites	Light source	morphology	pollutants	K_{app}	Ref.
Bi_2WO_6/ TiO_2-NT	350 W Xe λ > 420 nm	Bi_2WO_6 nanoparticles coated on the TiO_2 nanotubes	RhB<br>Cr(VI)	0.65 h^{-1}<br>0.52 h^{-1}	This work
Ag_2O-NP/ TiO_2-NT	300 W, Hg	Ag_2O nanoparticles coated on the TiO_2 nanotubes	MB<br>RhB<br>NFCX	0.62 min^{-1}<br>0.37 min^{-1}<br>0.29 min^{-1}	[1]
g-C_3N_4/ TiO_2-NT	350 W Xe λ > 420 nm	g-C_3N_4 layer anchored on the TiO_2 nanotube surfaces	MB	0.21 h^{-1}	[2]
$H_3PW_{12}O_{40}$/ TiO_2-NT	300 W, Hg	$H_3PW_{12}O_{40}$ nanoparticles coated on the TiO_2 nanotubes or on the TiO2/cellulose composite sheet	MB	0.54 min^{-1}	[3]

References

1. Lin, Z.; Lu, Y.; Huang, J. *Cellulose* **2019**, *26*, 6683−6700.

2. Lin, Z.; Yu, B.; Huang, J. *Langmuir* **2020**, *36*, 5967−5978.

3. Lin, Z.; Huang, J. *Sep. Purif. Technol.* **2021**, *264*, 118427.
